# ﻿Taxonomy and phylogeny of Irpicaceae and Meruliaceae (Polyporales, Basidiomycota) with descriptions of four new species from southwestern China

**DOI:** 10.3897/mycokeys.119.157018

**Published:** 2025-07-04

**Authors:** Zirui Gu, Chunqin Zhou, Jianling Zhang, Yu Liu, Qiaohua Deng, Rongcong Yang, Shunqiang Yang, Changlin Zhao

**Affiliations:** 1 The Key Laboratory of Forest Resources Conservation and Utilization in the South-west Mountains of China Ministry of Education, Key Laboratory of National Forestry and Grassland Administration on Biodiversity Conservation in Southwest China, Yunnan Provincial Key Laboratory for Conservation and Utilization of In-forest Re-source, Southwest Forestry University, Kunming 650224, China; 2 College of Forestry, Southwest Forestry University, Kunming 650224, China; 3 Yunnan Wumeng Mountains National Nature Reserve Administration Bureau, Zhaotong 657000, China; 4 Yunnan Tongbiguan Provincial Nature Reserve, Mangshi, 678400, China; 5 Yunnan Key Laboratory of Gastrodia and Fungal Symbiotic Biology, Zhaotong University, Zhaotong, 657000, China

**Keywords:** Four new species, molecular systematics, taxonomy, wood-inhabiting fungi, Yunnan Province

## Abstract

Wood-decaying fungi play crucial roles as decomposers in forest ecosystems. In this study, four new wood-inhabiting fungi from Yunnan Province of southwest China—*Crystallicutisalbomarginata*, *Efibulaglossophora*, *E.punctata*, and *Scopuloidesfarinacea*—are described and illustrated based on morphological and molecular evidence. The species *C.albomarginata* is characterized by its slightly pink to orange basidiomata when dry, a monomitic hyphal system with simple septa, and narrowly ellipsoid basidiospores measuring 3.7–4.4 × 1.9–2.8 µm. The taxon *E.glossophora* is characterized by its hard, membranous, slightly yellow to yellow basidiomata; a monomitic hyphal system with simple-septate generative hyphae; and ellipsoid basidiospores measuring 3.8–6 × 2.6–3.7 µm. The species *E.punctata* is characterized by its membranaceous, slightly gray to pale brown basidiomata; a monomitic hyphal system with simple-septate generative hyphae; and ellipsoid basidiospores measuring 4.3–6.2 × 2.2–3.3 µm. Additionally, the taxon *S. farinacea* is characterized by its coriaceous, pale cream to buff basidiomata; a monomitic hyphal system with simple-septate generative hyphae; and ellipsoid basidiospores measuring 2.8–3.5 × 1.4–2 µm. Sequences of the internal transcribed spacer region (ITS), the large subunit nuclear ribosomal RNA gene (nLSU), the translation elongation factor 1-α gene (TEF1), the largest subunit of RNA polymerase II (RPB1), and the second subunit of RNA polymerase II (RPB2) of the studied samples were employed, and phylogenetic analyses were performed using maximum likelihood, maximum parsimony, and Bayesian inference methods, ensuring the robustness of the findings. Based on the combined ITS + nLSU + RPB1 + RPB2 + TEF1 dataset, phylogenies of the two families Irpicaceae and Meruliaceae were constructed. In these analyses, *Crystallicutisalbomarginata* was recovered as sister to *C.serpens*; *E.punctata* as sister to *E.intertexta*; *Efibulaglossophora* as closely related to *E.intertexta* and *E.hainanensis*; and the new species *S. farinacea* as sister to *S. allantoidea*.

## ﻿Introduction

Fungi play vital roles in forest ecosystems as endophytes, pathogens, and saprobes (Wei and Dai 2004; Dai 2012; Cui et al. 2019; Bhunjun et al. 2022; Dong et al. 2024; Hyde et al. 2024; Yang et al. 2025a). The number of species in the kingdom Fungi is estimated to range from 2.2 to 13.2 million, with the latest estimate suggesting 2 to 3 million species. However, only around 150,000 species have been named and classified to date (Dai and Zhuang 2010; Bhunjun et al. 2022; He et al. 2024; Hyde et al. 2024; Zhao et al. 2024). Undescribed fungi therefore represent a vast potential resource at a time when there is an urgent need to discover new sources of food, antibiotics, and metabolites with biotechnological, industrial, and pharmaceutical applications (Hyde et al. 2019).

Species in the phlebioid clade of the Polyporales are wood-inhabiting fungi associated with white rot and typically produce crust-like or corticioid basidiomata with smooth or variously shaped hymenophores (Chen et al. 2021; Li et al. 2025). Within this clade, three well-supported subclades are recognized: the families Phanerochaetaceae Jülich, Irpicaceae Spirin & Zmitr., and Meruliaceae Rea. The ecology, species diversity, taxonomy, and molecular systematics of taxa in these families have been extensively studied (Floudas and Hibbett 2015; Miettinen et al. 2016; Justo et al. 2017; Zmitrovich 2018; Chen et al. 2021; Li et al. 2022, 2023; Lira et al. 2022; Motato-Vásquez et al. 2022; Wang et al. 2023; Yuan et al. 2023; Zhao et al. 2023; Xu et al. 2024; Li et al. 2025; Liu et al. 2025a).

In terms of morphology, the Irpicaceae comprises corticioid species or resupinate to pileate polypores. The hyphal system is usually monomitic, rarely dimitic; generative hyphae are typically simple-septate, rarely nodose-septate. Cystidia are often absent, and basidiospores are generally thin-walled, smooth, and colorless. The Meruliaceae includes corticioid species, resupinate or pileate polypores, or pileate hydnaceous species, which are often ceraceous in appearance. The hyphal system is usually monomitic, rarely dimitic, and tightly arranged; generative hyphae are usually nodose-septate, rarely simple-septate. Cystidia are often present, and basidiospores are typically thin-walled, smooth, and colorless (Justo et al. 2017; Chen et al. 2021; Li et al. 2025).

The genus *Efibula* Sheng H. Wu was introduced by Wu (1990) and typified by *E.tropica* Sheng H. Wu. This genus is characterized by membranaceous, subceraceous to ceraceous, buff-brownish basidiomata with smooth-tuberculate hymenophore, a subiculum of dense texture, a monomitic hyphal system with typically simple-septate hyphae, and an absence of cystidia. Cystidioles and hyphidia are present in some species (Chen et al. 2021). Species in *Efibula* are morphologically similar and can be distinguished only by slight variations in basidiospore size and shape, the presence of cystidioles and hyphidia, and hymenial configuration and color (Chen et al. 2021). Based on the MycoBank database (http://www.mycobank.org, accessed on 25 April 2025) and Index Fungorum (http://www.indexfungorum.org, accessed on 25 April 2025), the genus *Efibula* has registered 29 specific and infraspecific names, with 26 species widely recognized (Hyde et al. 2024).

Currently, DNA sequence-based classification and identification have become the standard in fungal taxonomy. Revisiting the taxonomy of *Phanerochaete* P. Karst. using a four-gene dataset revealed that four *Efibula* species clustered together and grouped with *Byssomeruliuscorium* (Pers.) Parmasto (Floudas and Hibbett 2015). A phylogenetic study revising the family-level classification of the Polyporales showed that *E.clarkiae* Floudas & Hibbett and *E.gracilis* Floudas & Hibbett grouped together, with *Efibula* species nested within Irpicaceae (Justo et al. 2017). Chen et al. (2021) demonstrated through multigene phylogenetic analyses that *Efibula* was paraphyletic in the phylogenetic tree, although there was insufficient morphological evidence to support the recognition of separate genera. Dong et al. (2024) introduced a new *Efibula* species, characterized morphologically and analyzed phylogenetically using ITS, nLSU, and TEF1 sequences.

The genus *Crystallicutis* El-Gharabawy, Leal-Dutra & G.W. Griff. was typified by *Crystallicutisdamiettensis* El-Gharabawy, Leal Dutra & G.W. Griff. (El-Gharabawy et al. 2021). *Crystallicutis* differs from other Irpicaceae members by its encrusted subicular hyphae and typically yellow hymenial surface when fresh. Basidiomata are resupinate with smooth, tuberculate, papillate, merulioid, or occasionally poroid hymenophores, usually honey-yellow (but occasionally rosy, reddish, or greenish), waxy, with white margins. Subicular hyphae, and sometimes the hymenium/subhymenium, are encrusted with crystals associated with darker resinous granules. The hyphal system is monomitic, usually with clamp connections. Basidia are cylindric-clavate; basidiospores are hyaline, smooth, ellipsoid, non-amyloid, and non-dextrinoid (El-Gharabawy et al. 2021). Based on MycoBank and Index Fungorum (accessed on 25 April 2025), the genus *Crystallicutis* has registered four specific and infraspecific names, with four species widely recognized (Hyde et al. 2024).

The taxonomy of Polyporales is complicated by the variability of key morphological characters across families and genera but is gradually being resolved through molecular phylogenetic analyses (El-Gharabawy et al. 2021). Multigene phylogenetic analyses based on ITS, LSU, TEF1, RPB1, and RPB2 loci placed *Crystallicutisdamiettensis* in Irpicaceae and formed a distinct clade with *Ceraceomycesserpens* (Tode) Ginns and several other previously unnamed taxa, thus establishing the new genus *Crystallicutis* (El-Gharabawy et al. 2021).

Meruliaceae was introduced by Rea in 1922 and has been strongly supported as a monophyletic clade in Polyporales. To date, 28 genera have been described in Meruliaceae (Floudas and Hibbett 2015; Justo et al. 2017; Chen et al. 2021; Li et al. 2025). *Scopuloides* (Massee) Höhn. & Litsch. forms a well-supported monophyletic group (Li et al. 2025). The genus *Scopuloides*, typified by *S. hydnoides* (Cooke & Massee) Hjortstam & Ryvarden, has been recovered as monophyletic within Meruliaceae and includes species from Asia, Europe, North America, and the Neotropics (Chen et al. 2021). *Scopuloides* species are typically ceraceous, with white to buff basidiomata, and odontioid, hydnoid, or grandinioid hymenophores. The genus is characterized by a compact subiculum with agglutinated, short-celled subicular hyphae, short basidia, small basidiospores, and the presence of lamprocystidia (Wu 1990; Gilbertson and Nakasone 2003; Bernicchia and Gorjón 2010). Based on MycoBank and Index Fungorum (accessed on 25 April 2025), *Scopuloides* has registered 15 specific and infraspecific names, with 10 species widely recognized (Hyde et al. 2024).

Species diversity, taxonomy, and multigene phylogeny of the phlebioid clade (Phanerochaetaceae, Irpicaceae, Meruliaceae) of Polyporales Gäum. have shown that four species of *Scopuloides* grouped together based on nuc rDNA ITS1-5.8S-ITS2, 28S rDNA, RNA polymerase II largest subunit (RPB1), RNA polymerase II second largest subunit (RPB2), and translation elongation factor 1-α (TEF1), with all species clustering within Meruliaceae (Chen et al. 2021). Multigene phylogeny and taxonomy of the wood-inhabiting fungal genus *Phlebia* sensu lato Fr. (Polyporales, Basidiomycota) indicated that four *Scopuloides* taxa were nested within Meruliaceae and closely related to *Climacodon* P. Karst. and *Luteochaete* C.C. Chen & Sheng H. Wu, based on ITS+nLSU+TEF1+mtSSU+GAPDH+RPB1+RPB2 sequences (Zhao et al. 2023). Phylogenetic analyses were carried out on two datasets—ITS+nLSU and five-gene (ITS+nLSU+RPB1+RPB2+TEF1) sequences—to further explore species diversity in Meruliaceae, clarify the validity and circumscription of select genera, and contribute to a well-supported and robust phylogeny of the family (Li et al. 2025).

During investigations of wood-inhabiting fungi in Yunnan Province, China, four undescribed species were discovered and are introduced herein. The aims of this study are to confirm the taxonomic affinities of the new species and to carry out a phylogenetic and taxonomic study based on ITS, nLSU, RPB1, RPB2, and TEF1 sequences to elucidate the relationships among representative species of Irpicaceae and Meruliaceae. Accordingly, four new species—*Crystallicutisalbomarginata*, *Efibulaglossophora*, *E.punctata*, and *Scopuloidesfarinacea*—are proposed, with descriptions and illustrations based on morphological characteristics and phylogenetic analyses.

## ﻿Materials and methods

### ﻿Sample collection and fungarium specimen preparation

Basidiomata were collected from Yunnan Province, China. Fresh specimens were photographed in situ using a Nikon D7100 digital camera, and macroscopic features were recorded in the field. All photographs were focus-stacked using Helicon Focus software. Specimens were then transported to a field station and dried using an electric food dryer at 45 °C. Once fully dried, the samples were sealed in envelopes and zip-lock plastic bags, labeled, and deposited in the fungarium of the Southwest Forestry University (SWFC), Kunming, Yunnan Province, China.

### ﻿Morphology

The macromorphological descriptions were based on field notes and photos taken *in situ* and in the laboratory. The macro-morphology was based on both fresh and dried specimens. The color terms used in the descriptions followed Anonymous and Petersen (Anonymous 1969; Petersen 1996). Micromorphological data were obtained from the dried specimens and observed under a light microscope following previous studies (Dai 2010; Wu et al. 2022a). Sections were mounted in 5% KOH and 2% phloxine B dye (C_20_H_2_Br_4_Cl_4_Na_2_O_5_), and other reagents, including cotton blue and Melzer’s reagent, were also used to observe micromorphology following Wu et al. (2022a). To show variation in spore sizes, 5% of measurements were excluded from each end of the range and shown in parentheses. At least thirty basidiospores from each specimen were measured. Stalks were excluded from basidia measurements, and the hilar appendage was excluded from basidiospore measurements. The following abbreviations were used: CB = cotton blue, CB– = acyanophilous, KOH = 5% potassium hydroxide, IKI = Melzer’s reagent, IKI– = both inamyloid and indextrinoid. W = mean spore width (arithmetic average for all spores), L = mean spore length (arithmetic average for all spores), Q = variation in the L/W ratios between the specimens studied, and n (a/b) = number of spores (a) measured from a given number (b) of specimens (Guan and Zhao 2021; Wu et al. 2022a; Wang et al. 2023).

### ﻿Molecular phylogeny

Genomic DNA was extracted from dried basidiomata using a cetyltrimethylammonium bromide rapid plant genome extraction kit (Aidlab Biotechnologies Co., Ltd., Beijing) according to the manufacturer’s instructions: a small piece of dried fungal specimen (about 30 mg) was ground to powder with liquid nitrogen. The powder was transferred to a 1.5 mL centrifuge tube, suspended in 0.4 mL of lysis buffer, and incubated at 65 °C in a water bath for 60 min. After that, 0.4 mL of phenol-chloroform (24:1) was added to each tube, and the suspension was shaken vigorously. After centrifugation at 13,000 rpm for 5 min, 0.3 mL of supernatant was transferred to a new tube and mixed with 0.45 mL of binding buffer. The mixture was then transferred to an adsorbing column (AC) for centrifugation at 13,000 rpm for 0.5 min. Then, 0.5 mL of inhibitor removal fluid was added to the AC for centrifugation at 12,000 rpm for 0.5 min. After washing twice with 0.5 mL of washing buffer, the AC was transferred to a clean centrifuge tube, and 100 µL of elution buffer was added to the middle of the adsorbed film to elute the genomic DNA (Zhao et al. 2012). The primer pair ITS5 and ITS4 (White et al. 1990) was used to amplify the ITS region. The primer pair LR0R and LR7 (Vilgalys and Hester 1990; Rehner and Samuels 1994; Yang et al. 2025b) was used to amplify the nuclear LSU region. The RPB1 region was initially amplified with RPB1-Af and RPB1-Cf (Matheny et al. 2002). RPB2 was amplified with the primer pair bRPB2-6F and bRPB2-7.1R (Liu et al. 1999; Matheny et al. 2002; Matheny 2005). TEF1 was amplified with the primer pair EF1-983F and EF1-2218R (Dong et al. 2024). PCR was performed following Dong et al. (2024). All newly generated sequences were deposited in the GenBank database. All sequences are listed in Table 1.

**Table 1.** List of species, specimens and GenBank accession numbers of sequences used in this study. [New species is in bold; * is type material, holotype].

Species name	Specimen No.	GenBank accession No.					Country	References
		ITS	nLUS	*rpb1*	*rpb2*	*tef1-α*		
*Allophlebiaformosana*	He 3805	PP549545	PP549577	—	—	—	China	Li et al. (2025)
*Allophlebiaformosana*	He 4394	PP549546	PP549578	—	—	—	China	Li et al. (2025)
*Aurantiopileusmayanensis*	JV 1504/128	KT156706	—	—	—	—	Costa Rica	Zhao et al. (2023)
*Aurantiopileusmayaennsis*	TJB10228	HM772140	HM772139	—	—	—	Belize	Ginns et al. (2010)
*Aurantiporusvenustus*	391/12	OL630489	OL635577	—	—	—	Brazil	Unpublished
*Bjerkanderaadusta*	HHB-12826-Sp	KP134983	KP135198	—	—	—	USA	El-Gharabawy et al. (2021)
*Bjerkanderaadusta*	MUT<ITA>:5195	KM355986	MF115840	—	—	—	Italy	El-Gharabawy et al. (2021)
*Byssomeruliuscorium*	FP-102382	KP135007	KP135230	KP134802	KP134921	—	USA	Floudas and Hibbett (2015)
*Byssomeruliuscorium*	WEI 17-645	LC427006	LC427030	—	—	—	China	Chen et al. 2020
*Candelabrochaeteguangdongensis*	He 5902	MZ422527	MZ422499	—	—	—	China	Li et al. (2022)
*Ceriporiamellita*	GC 1508-71	LC427022	LC427044	—	—	—	China	Chen et al. (2021)
*Ceriporiamellita*	WEI 17-024	LC427024	LC427046	—	—	—	China	Chen et al. (2021)
*Ceriporiaviridans*	GC 1708-211	LC427027	LC427049	LC427062	—	—	China	Chen et al. (2021)
*Ceriporiaviridans*	Miettinen 11701	KX752600	KX752600	—	—	—	Netherlands	Miettinen et al. (2016)
*Ceriporiopsistianshanensis*	Cui 19150	OP920992	OP920984	—	—	—	China	Xu et al. (2023)
*Ceriporiopsistianshanensis*	Cui 19151	OP920993	OP920985	—	—	—	China	Xu et al. (2023)
*Ceriporiopsoidesguidella*	HUBO 7659	FJ496687	FJ496722	—	—	—	Italy	Tomšovský et al. (2010)
*Ceriporiopsoideslagerheimii*	58240	KX008365	KX081077	—	—	—	China	Zhao et al. (2017)
*Ceriporiopsoideslagerheimii*	Dai 12304	KX161647	KX161651	—	—	—	China	Zhao et al. (2023)
*Climacodonseptentrionalis*	AFTOL‐767	AY854082	AY684165	AY864873	AY780941	AY885151	USA	Lutzoni et al. (2004)
*Climacodonseptentrionalis*	RLG-6890-Sp	KP135344	—	—	—	—	USA	Floudas and Hibbett (2015)
*Crustodontiachrysocreas*	HHB‐6333‐Sp	KP135358	KP135263	KP134861	KP134908	—	USA	Floudas and Hibbett (2015)
*Crustodontianigrodontea*	CLZhao 2758	MT896824	—	—	—	—	China	Huang et al. (2020)
***Crystallicutisalbomarginata***	**CLZhao 31409**	**PV470538**	**PV474183**	—	—	**PV759048**	**China**	**Present study**
***Crystallicutisalbomarginata***	**CLZhao 31506** *	**PV470539**	**PV474184**	—	**PV759033**	**PV759049**	**China**	**Present study**
*Crystallicutisdamiettensis*	UN63A	KX428470	MW508515	MW523003	—	MW523002	Egypt	El-Gharabawy et al. (2016)
*Crystallicutishuangshanensis*	Dai 6090	JX623934	JX644066	—	—	—	China	El-Gharabawy et al. (2021)
*Crystallicutisrajchenbergii*	MR-4310	KY948797	KY948888	KY948963	—	—	USA	Justo et al. (2017)
*Crystallicutisserpens*	HHB-15692-Sp	KP135031	KP135200	KP134785	KP134914	—	USA	Floudas and Hibbett (2015)
*Crystallicutisserpens*	TNM:F30569	MZ636946	—	—	—	—	China	Chen et al. (2021)
*Cytidiellaalbomarginata*	He 5575	MZ422526	MZ422497	—	—	—	China	Li et al. (2022)
*Cytidiellaalbomarginata*	WEI 18-474	MZ636948	MZ637110	—	—	—	China	Chen et al. (2021)
*Cytidiellanitidula*	He 5126	MZ422523	MZ422494	—	—	—	China	Li et al. (2022)
*Cytidiellanitidula*	He 5135	MZ422524	MZ422495	—	—	—	China	Li et al. (2022)
*Efibulaamericana*	HHB-10209-Sp	KP135014	—	—	—	—	USA	Floudas and Hibbett (2015)
*Efibulaamericana*	FP-102165	KP135016	KP135256	KP134808	KP134916	MZ913669	USA	Floudas and Hibbett (2015)
*Efibulaamericana*	HHB-8468	KP135012	—	—	—	—	USA	Floudas and Hibbett (2015)
*Efibulaclarkii*	FD-228	KP135019	—	KP134803	—	—	USA	Floudas and Hibbett (2015)
*Efibulacordylines*	ICMP 18129	PQ214293	—	—	—	—	New Zealand	Unpublished
*Efibuladaweishanensis*	CLZhao 18946	OR094488	—	—	—	OR541912	China	Dong et al. (2024)
*Efibuladaweishanensis*	CLZhao 19002	OR094489	OR449958	—	—	—	China	Dong et al. (2024)
*Efibuladaweishanensis*	CLZhao 25072	OR094490	OR449959	—	OR733284	OR541913	China	Dong et al. (2024)
***Efibulaglossophora***	**CLZhao 22744** *	**PV470540**	**PV474185**	**PV747409**	**PV759036**	**PV759050**	**China**	**Present study**
*Efibulagracilis*	FD-455	KP135027	MZ637116	KP134804	OK136077	MZ913679	USA	Chen et al. (2021)
*Efibulagracilis*	FP-102052	KP135028	—	—	—	—	USA	Floudas and Hibbett (2015)
*Efibulagrandinosa*	He 6312	MZ422509	MZ422480	—	—	—	China	Li et al. (2022)
*Efibulahainanensis*	He 6004	MW580949	MW580939	—	—	—	China	Li et al. (2022)
*Efibulahainanensis*	Chen 1284	ON117184	—	—	—	—	China	Li et al. (2022)
*Efibulaintertexta*	Wu 1707-93	MZ636953	MZ637117	MZ748416	OK136085	—	China	Chen et al. (2021)
*Efibulaintertexta*	Wu 1707-96	MZ636954	MZ637118	MZ748417	OK136086	—	China	Chen et al. (2021)
*Efibulamatsuensis*	Wu 1011-18	MZ636956	MZ637119	MZ748418	OK136078	MZ913680	China	Chen et al. (2021)
*Efibulamatsuensis*	Wu 1011-19	MZ636957	MZ637120	—	—	—	China	Chen et al. (2021)
***Efibulapunctata***	**CLZhao 22764**	**PV470541**	**PV474186**	**PV747410**	**PV759037**	**PV759051**	**China**	**Present study**
***Efibulapunctata***	**CLZhao 29674**	**PV470542**	**PV474187**	—	**PV759038**	**PV759052**	**China**	**Present study**
***Efibulapunctata***	**CLZhao 29678**	**PV470543**	**PV474188**	**PV747411**	**PV759039**	**PV759053**	**China**	**Present study**
***Efibulapunctata***	**CLZhao 30011** *	**PV470544**	**PV474189**	—	**PV759040**	**PV759054**	**China**	**Present study**
***Efibulapunctata***	**CLZhao 30054**	**PV470545**	**PV474190**	**PV747412**	**PV759041**	**PV759055**	**China**	**Present study**
***Efibulapunctata***	**CLZhao 30615**	**PV470546**	**PV474191**	—	—	**PV759056**	**China**	**Present study**
***Efibulapunctata***	**CLZhao 30637**	**PV470547**	**PV474192**	**PV747413**	**PV759042**	**PV759057**	**China**	**Present study**
***Efibulapunctata***	**CLZhao 30648**	**PV470548**	**PV474193**	—	**PV759043**	**PV759058**	**China**	**Present study**
***Efibulapunctata***	**CLZhao 30659**	**PV470549**	**PV474194**	**PV747414**	**PV759044**	**PV759059**	**China**	**Present study**
***Efibulapunctata***	**CLZhao 30664**	**PV470550**	**PV474195**	**PV747415**	**PV759045**	**PV759060**	**China**	**Present study**
***Efibulapunctata***	**CLZhao 30691**	**PV470551**	**PV474196**	**PV747416**	**PV759046**	**PV759061**	**China**	**Present study**
***Efibulapunctata***	**CLZhao 30718**	**PV470552**	**PV474197**	**PV747417**	**PV759047**	**PV759062**	**China**	**Present study**
*Efibulashenghuae*	He 3384	MZ422508	MZ422479	—	—	—	China	Li et al. (2022)
*Efibulasubglobispora*	He 3983	MW580944	MW580934	—	—	—	China	Li et al. (2022)
*Efibulasubglobispora*	He 7032	MZ422506	MZ422477	—	—	—	China	Li et al. (2022)
*Efibulasubglobispora*	Chen 1716	MZ636962	MZ637124	MZ748427	OK136075	MZ913673	China	Chen et al. (2021)
*Efibulasubglobispora*	GC 1604-13	MZ636963	MZ637125	MZ748428	OK136076	MZ913674	China	Chen et al. (2021)
*Efibulataiwanensis*	He 4582	MZ422507	MZ422478	—	—	—	China	Li et al. (2022)
*Efibulatropica*	WEI 18-149	MZ636967	MZ637129	MZ748419	OK136079	MZ913681	China	Chen et al. (2021)
*Efibulatropica*	Wu 0809-8	MZ636968	MZ637130	—	—	—	China	Chen et al. (2021)
*Efibulatuberculata*	Wu 1005-55	MZ636970	MZ637132	MZ748426	OK136074	MZ913672	China	Chen et al. (2021)
*Efibulatuberculata*	Wu 0711-148	MZ636969	MZ637131	—	—	MZ913671	China	Chen et al. (2021)
*Efibulaturgida*	Wu 0910-86	MZ636972	MZ637134	MZ748439	OK136091	MZ913716	China	Chen et al. (2021)
*Efibulaturgida*	Wu 0910-99	MZ636973	MZ637135	MZ748440	OK136092	MZ913717	China	Chen et al. (2021)
*Efibulayunnanensis*	He 4653	MW580948	MW580938	—	—	—	China	Li et al. (2022)
*Efibulayunnanensis*	He 6970	MZ422505	MZ422476	—	—	—	China	Li et al. (2022)
*Efibulayunnanensis*	Wu 880515-1	MZ636977	GQ470672	MZ748420	OK136080	MZ913682	China	Chen et al. (2021)
*Efibulayunnanensis*	CLZhao 11641	MT611529	—	—	—	—	China	Ma et al. (2020)
*Flavodonambrosius*	HULCR-6860	KR119074	KR119077	—	—	—	USA	El-Gharabawy et al. (2021)
*Flavodonflavus*	LE295997	KF856505	KF856510	—	—	—	Tanzania	Zmitrovich and Malysheva (2014)
*Flavodonflavus*	WHC 1381	LC427029	LC427052	—	—	—	China	Chen et al. (2021)
*Geesteraniacarneola*	MCW 388/12	KY174999	KY174999	—	KY175011	KY175013	Brazil	Westphalen et al. (2018)
*Geesteraniadavidiae*	MCW 396/12	KY174998	KY174998	—	KY175012	KY175016	Brazil	Westphalen et al. (2018)
*Gloeoporushainanensis*	Yuan-4397	KU360400	KU360409	—	—	—	China	El-Gharabawy et al. (2021)
*Gloeoporuspannocinctus*	L-15726-Sp	KP135060	KP135214	KP134867	KP134973	—	USA	El-Gharabawy et al. (2021)
*Hermanssoniacentrifuga*	CBS 125890	MH864088	MH875547	—	—	—	Sweden	Vu et al. (2019)
*Hermanssoniacentrifuga*	HHB-9239-Sp	KP135380	KP135262	KP134844	KP134974	MZ913721	USA	Floudas and Hibbett (2015)
*Hermanssoniafimbriata*	Dai 23266	ON135436	ON135440	—	—	—	China	Liu et al. (2022)
*Hermanssoniafimbriata*	Dai 23305	ON135437	ON135441	—	—	—	China	Liu et al. (2022)
*Hydnophanerochaeteodontoidea*	CLZhao 3882	MH784919	MH784929	—	—	—	China	Shen et al. (2018)
*Hydnophanerochaeteodontoidea*	CLZhao 4036	MH784927	MH784937	—	—	—	China	Shen et al. (2018)
*Hydnophlebiaacanthocystis*	FP 150571	KY948767	KY948844	KY948914	—	—	USA	Justo et al. (2017)
*Hydnophlebiachrysorhiza*	FD‐282	KP135338	KP135217	KP134848	KP134897	—	USA	Floudas and Hibbett (2015)
*Hydnophlebiachrysorhiza*	HHB-18767	KP135337	—	—	—	—	USA	Floudas and Hibbett (2015)
*Hydnophlebiagorgonea*	MA‐Fungi 86642	KF483031	KF528122	—	—	—	Cape Verde	Telleria et al. (2017)
*Hyphodermasetigerum*	FD-312	KP135297	KP135222	KP134871	—	—	USA	Floudas and Hibbett (2015)
*Irpexalboflavescens*	He 3933	MZ422503	MZ422474	—	—	—	China	Li et al. (2022)
*Irpexalboflavescens*	He 4719	MZ422501	MZ422472	—	—	—	China	Li et al. (2022)
*Irpexlatemarginatus*	FP-55521T	KP135024	KP135202	KP134805	KP134915	—	USA	Floudas and Hibbett (2015)
*Irpexlatemarginatus*	Piatek 1997	KX752592	KX752592	—	—	—	Poland	Miettinen et al. (2016)
*Irpexrosea*	He 6277	MW580943	MW580933	—	—	—	China	Li et al. (2022)
*Irpexrosea*	CLZhao 18491	MW377575	MW377578	—	—	—	China	Wang and Zhao (2022)
*Leptoporusmollis*	RLG-7163	KY948794	—	KY948956	—	—	USA	Justo et al. (2017)
*Leptoporusmollis*	TJV_93_174T	KY948795	EU402510	KY948957	—	—	USA	Justo et al. (2017)
*Lilaceophlebiatremelloidea*	Bosco_Siro_Negri_1	PP716904	PP716909	—	—	—	Italy	Girometta et al. (2025)
*Lilaceophlebiatremelloidea*	Bosco_Siro_Negri_2	PP716903	PP716910	—	—	—	Italy	Girometta et al. (2025)
*Luteochaetesubglobosa*	CLZhao 3639	MK881898	MK881788	—	—	—	China	Huang et al. (2020)
*Luteochaetesubglobosa*	CLZhao 3475	MK881897	MK881787	—	—	—	China	Huang et al. (2020)
*Luteoporiaalbomarginata*	GC 1702-1	LC379003	LC379155	LC379160	LC387358	LC387377	China	Wu et al. (2016)
*Luteoporiacitriniporia*	Dai 19507	MT872218	MT872216	—	—	—	China	Liu and Yuan (2020)
*Luteoporiastraminea*	CLZhao 18947	MW732407	MW724799	—	—	—	China	Zhao et al. (2023)
*Meruliopsiscrassitunicata*	CHWC 1506-46	LC427010	LC427034	—	—	—	China	Chen et al. (2021)
*Meruliopsisleptocystidiata*	Wu 1708-43	LC427013	LC427033	—	—	—	China	Chen et al. (2021)
*Meruliopsisparvispora*	Wu 1209-58	LC427017	LC427039	—	—	—	China	Chen et al. (2021)
*Meruliopsistaxicola*	GC 1704-60	LC427028	LC427050	—	—	—	China	Chen et al. (2021)
*Meruliusnantahaliensis*	HHB-2816-sp	KY948777	KY948852	KY948920	—	—	USA	Justo et al. (2017)
*Meruliussinensis*	CLZhao 2562	MW732401	MW724793	—	—	—	China	Zhao et al. (2023)
*Mycoaciaaurea*	DLL 2011-263	KJ140747	—	—	—	—	USA	Binder et al. (2005)
*Mycoaciaaurea*	RLG 5075sp	KY948759	MZ637161	KY948918	—	—	USA	Binder et al. (2005)
*Mycoaciakunmingensis*	CLZhao 152	KX081072	KX081074	—	—	—	China	Zhao and Wu (2017)
*Mycoaciakunmingensis*	CLZhao 153	KX081073	KX081075	—	—	—	China	Zhao and Wu (2017)
*Mycoaciellaefibulata*	WEI 19-057	MZ637012	MZ637172	—	—	—	China	Chen et al. (2021)
*Mycoaciellaefibulata*	WEI 16-172	MZ637011	MZ637171	—	—	—	China	Chen et al. (2021)
*Odoriaalborubescens*	BP 106943	MG097864	MG097867	—	—	—	Hungary	Papp and Dima (2017)
*Odoriaalborubescens*	BRNU 627479	JQ821319	JQ821318	—	—	—	Czech Republic	Dvoˇrák et al. (2014)
*Pappiafissilis*	BRNM 699803	HQ728292	HQ729002	—	—	—	Czech Republic	Tomšovský (2012)
*Pappiafissilis*	814	HQ728291	HQ729001	—	—	—	Czech Republic	Tomšovský (2012)
*Phanerochaetellaformosana*	He 3391	MZ422520	MZ422491	—	—	—	China	Li et al. (2022)
*Phanerochaetellaformosana*	He 3962	MZ422522	MZ422493	—	—	—	China	Li et al. (2022)
*Phanerochaetellaqueletii*	He 3050	MZ422512	MZ422483	—	—	—	China	Li et al. (2022)
*Phanerochaetellaqueletii*	He 3284	MZ422510	MZ422481	—	—	—	China	Li et al. (2022)
*Phlebiaacerina*	FD 301	KP135378	—	KP134862	—	—	USA	Justo et al. (2017)
*Phlebiaacerina*	HHB 11146	KP135372	—	—	—	—	USA	Floudas and Hibbett (2015)
*Phlebiafloridensis*	HHB‐9905-sp	KP135383	KP135264	KP134863	KP134899	—	USA	Floudas and Hibbett (2015)
*Phlebiaradiata*	CBS 285.56	MH857642	MH869187	—	—	—	France	Vu et al. (2019)
*Phlebiarufa*	FBCC297	LN611092	LN611092	—	—	—	Finland	Kuuskeri et al. (2015)
*Phlebiarufa*	HHB-14924	KP135374	—	—	—	—	USA	Floudas and Hibbett (2015)
*Phlebicolorataalboaurantia*	Cui 4136	KF845955	KF845948	—	—	—	China	Zhao and Cui (2014)
*Phlebicoloratapseudoplacenta*	Miettinen 18997	KY948744	KY948902	KY948926	—	—	USA	Justo et al. (2017)
*Phlebiodontiacaspica*	FCUG 3159	HQ153410	—	—	—	—	Iran	Ghobad-Nejhad and Hallenberg (2012)
*Phlebiodontiafissurata*	EM 13468	OR822135	—	—	—	—	Switzerland	Larsson et al. (2025)
*Phlebiporiabubalina*	Dai 13168	KC782526	KC782528	—	—	—	China	Chen and Cui (2014)
*Phlebiporiabubalina*	Dai 15179	KY131843	KY131902	—	—	—	China	Wu et al. (2017)
*Physisporinusvitreus*	Larsson 11959	JN710580	JN710580	—	—	—	Norway	Miettinen et al. (2012)
*Physisporinusyunnanensis*	CLZhao 21583	OP852341	OP852343	—	—	—	China	Cai et al. (2023)
*Physisporinusyunnanensis*	CLZhao 21647	OP852340	OP852342	—	—	—	China	Cai et al. (2023)
*Pseudonadsoniellabrunnea*	KTA-37	KT456204	—	—	—	—	Argentina	Kondratyuk et al. (2015)
*Pseudophlebiasetulosa*	HHB‐6891‐Sp	KP135382	KP135267	KP134864	KP134901	MZ913650	USA	Zong and Zhao (2021)
*Pseudophlebiasetulosa*	PH 11749	GU461312	GU461312	—	—	—	Spain	Moreno et al. (2011)
*Raduliporusaneirinus*	HHB-15629-Sp	KP135023	KP135207	KP134795	—	—	USA	Floudas and Hibbett (2015)
*Resiniporusresinascens*	BRNM 710169	FJ496675	FJ496698	—	—	—	Czech Republic	Tomšovský et al. (2010)
*Sarcodontiaamplissima*	CFMR:FP-104176	MZ322829	MZ322839	—	—	—	USA	Nakasone et al. (2021)
*Sarcodontiaamplissima*	CFMR:FP-101997	MZ322828	MZ322838	—	—	—	USA	Nakasone et al. (2021)
*Scopuloidesallantoidea*	GC 1602-11	MZ637080	MZ637278	—	—	—	China	Chen et al. (2021)
*Scopuloidesallantoidea*	WEI 16-060	MZ637081	MZ637279	—	OK136047	MZ913664	China	Chen et al. (2021)
*Scopuloidesdimorpha*	FP‐102935‐Sp	KP135353	KP135285	KP134855	KP134905	—	USA	Zong and Zhao (2021)
*Scopuloidesdimorpha*	WEI 19-073	MZ637084	MZ637282	—	—	—	China	Chen et al. (2021)
*Scopuloidesellipsoidea*	He 4681	PP549568	PP549602	—	—	—	China	Li et al. (2025)
*Scopuloidesellipsoidea*	He 4760	PP549569	PP549603	—	—	—	China	Li et al. (2025)
***Scopuloidesfarinacea***	**CLZhao 30005**	**PV470558**	**PV474203**	—	**PV759034**	**PV763684**	**China**	**Present study**
***Scopuloidesfarinacea***	**CLZhao 30181** *	**PV470559**	**PV474204**	—	—	**PV763685**	**China**	**Present study**
***Scopuloidesfarinacea***	**HMZhou 153**	**PV470560**	**PV474205**	—	**PV759035**	**PV763686**	**China**	**Present study**
*Scopuloidesgrandinioides*	He 6295	PP549571	PP549605	—	PP566657	—	China	Li et al. (2025)
*Scopuloidesgrandinioides*	RLG-5104-sp	KP135351	KP135283	—	KP134904	—	USA	Li et al. (2025)
*Scopuloideshydnoides*	FP‐150473	KP135355	KP135284	KP134854	—	—	USA	Zong and Zhao (2021)
*Scopuloideshydnoides*	He 4507	PP549572	PP549606	—	—	—	China	Li et al. (2025)
*Scopuloidesleprosa*	FRDBI 17584773	MW487975	—	—	—	—	UK	Unpublished
*Scopuloidesrimosa*	HHB‐7042‐Sp	KP135350	KP135282	KP134853	KP134903	—	USA	Zong and Zhao (2021)
*Scopuloidesrimosa*	He 3320	PP549574	PP549608	—	—	—	China	Li et al. (2025)
*Scopuloidesrimosa*	He 3620	PP549575	PP549609	—	—	—	China	Li et al. (2025)
*Scopuloidesyunnanensis*	CLZhao 18588	PP511312	PP511315	—	—	—	China	Gu et al. (2024)
*Scopuloidesyunnanensis*	CLZhao 30079	PP511313	PP511316	—	—	—	China	Gu et al. (2024)
*Scopuloidesyunnanensis*	CLZhao 30213	PP511314	PP511317	—	—	—	China	Gu et al. (2024)
*Stereophlebiatuberculata*	FCUG 3157	HQ153427	—	—	—	—	Iran	Ghobad-Nejhad and Hallenberg (2012)
*Stereophlebiatuberculata*	Wu 1708-107	MZ637089	MZ637286	—	—	—	China	Chen et al. (2021)
*Trametopsisaborigena*	Robledo 1236	KY655336	KY655338	—	—	—	Argentina	Gómez-Montoya et al. (2017)
*Trametopsiscervina*	TJV-93-216T	JN165020	JN164796	JN164839	JN164877	JN164882	USA	Justo and Hibbett (2011)

**Figure 1.**
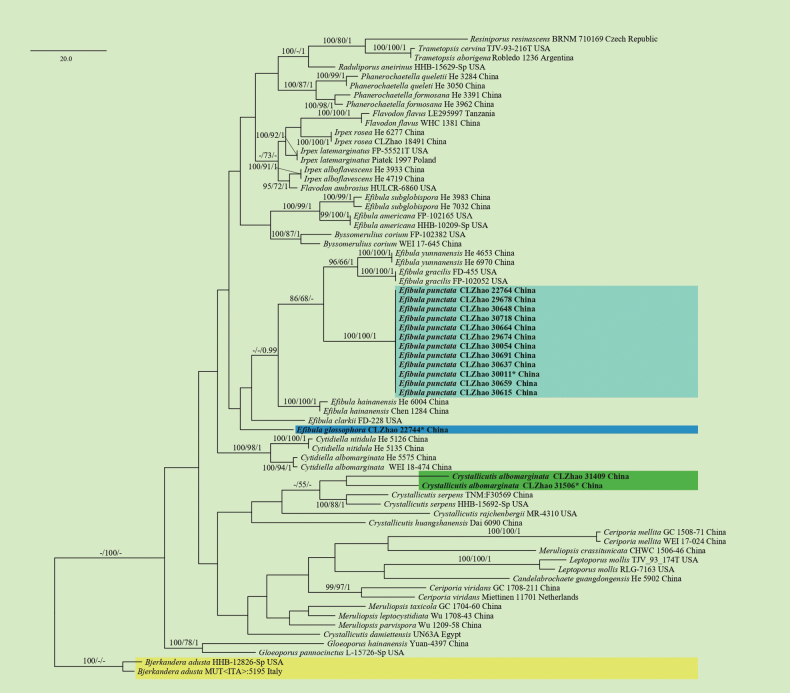
Maximum parsimony strict consensus tree illustrating the phylogenetic relationships of the three new species and related species within the family Irpicaceae based on ITS+nLSU+RPB1+RPB2+TEF1 sequences. Branches are labelled with maximum likelihood bootstrap value ≥ 70%, parsimony bootstrap value ≥ 50%, and Bayesian posterior probabilities ≥ 0.95. The new species is in bold.

**Figure 2.**
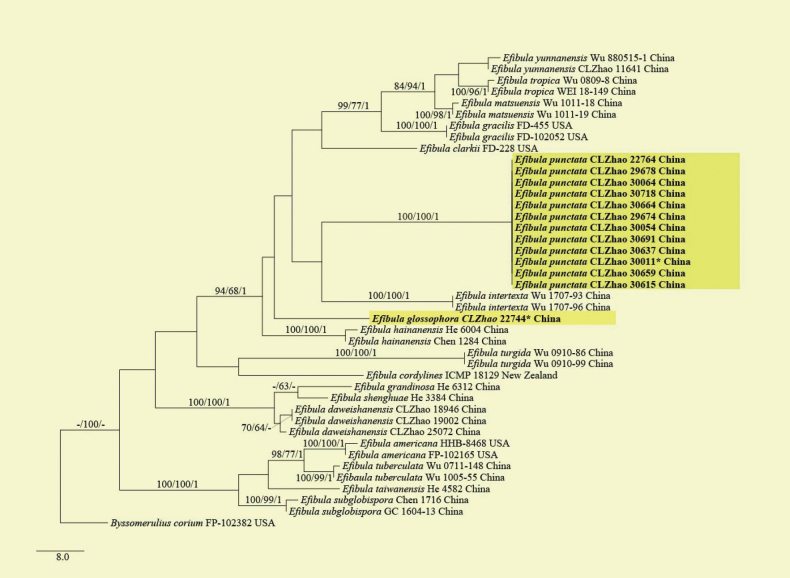
Maximum parsimony strict consensus tree illustrating the phylogenetic relationships of the three new species and related species in *Efibula* based on ITS+nLSU+RPB1+RPB2+TEF1 sequences. Branches are labelled with maximum likelihood bootstrap value ≥ 70%, parsimony bootstrap value ≥ 50%, and Bayesian posterior probabilities ≥ 0.95. The new species is in bold.

**Figure 3.**
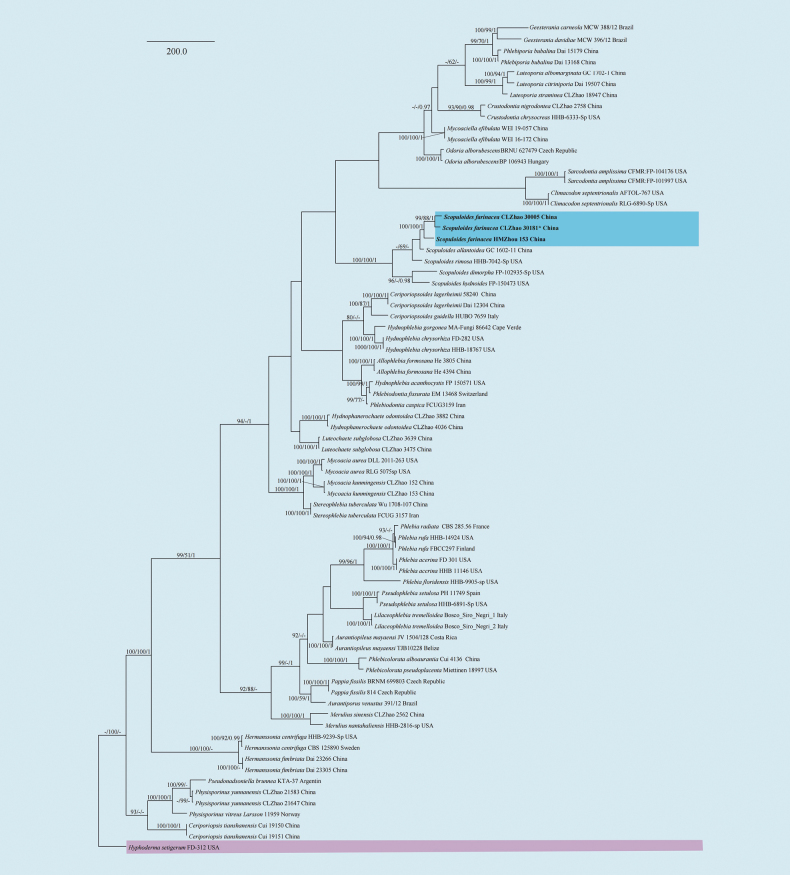
Maximum parsimony strict consensus tree illustrating the phylogenetic relationships of the new species *Scopuloidesfarinacea* and related species within the family Meruliaceae based on ITS+nLSU+RPB1+RPB2+TEF1 sequences. Branches are labelled with maximum likelihood bootstrap value ≥ 70%, parsimony bootstrap value ≥ 50%, and Bayesian posterior probabilities ≥ 0.95. The new species is in bold.

**Figure 4.**
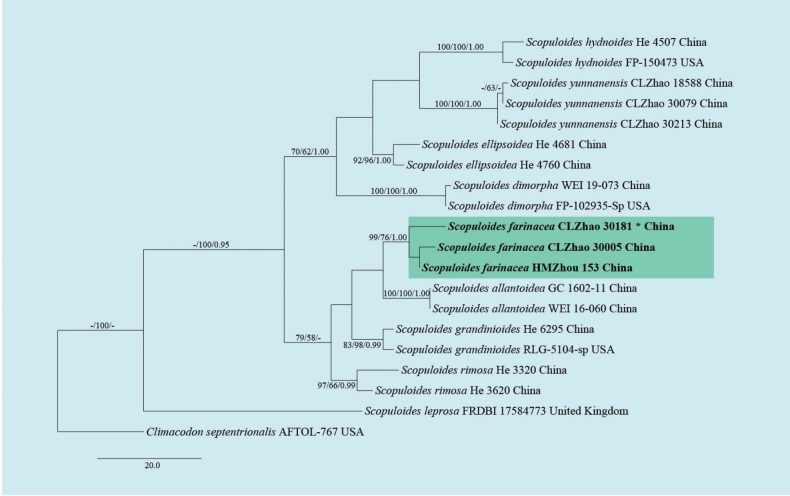
Maximum parsimony strict consensus tree illustrating the phylogenetic relationships of the new species *Scopuloidesfarinacea* and related species in *Scopuloides* based on ITS+nLSU+RPB2+TEF1 sequences. Branches are labelled with maximum likelihood bootstrap value ≥ 70%, parsimony bootstrap value ≥ 50%, and Bayesian posterior probabilities ≥ 0.95. The new species is in bold.

The sequences were aligned initially by using MAFFT (https://mafft.cbrc.jp/alignment/server/) using the “G-INS-I” strategy and then manually optimized in BioEdit and Aliview version 1.27 (Hall 1999; Yang et al. 2025b). The dataset is aligned first, and then ITS, nLSU, RPB1, RPB2, and TEF1 sequences are combined with Mesquite version 3.51. To determine the phylogeny, we compiled an ITS+nLSU+RPB1+RPB2+TEF1 sequence matrix, and *Bjerkanderaadusta* (Willd.) P. Karst. was used as an outgroup for phylogenetic analysis of the phylogenetic tree (Fig. 1). And *Byssomeruliuscorium* (Pers.) Parmasto was used as an outgroup for phylogenetic analysis of the ITS+nLSU+RPB1+RPB2+TEF1 phylogenetic tree (Fig. 2). To determine the phylogeny, we compiled an ITS+nLSU+RPB1+RPB2+TEF1 sequence matrix, and *Hyphodermasetigerum* (Fr.) Donk was used as an outgroup for phylogenetic analysis of the phylogenetic tree (Fig. 3). And *Climacodonseptentrionalis* (Fr.) P. Karst. was used as an outgroup for phylogenetic analysis of the ITS+nLSU+RPB2+TEF1 phylogenetic tree (Fig. 4).

Maximum parsimony (MP), maximum likelihood (ML), and Bayesian inference (BI) analyses were applied to the combined four datasets. The approach followed the previous study by Yang et al. (2025a) and Qin et al. (2025), and the tree construction procedure was performed in PAUP* version 4.0a169 (Swofford 2002; Yang et al. 2025b). All characters were equally weighted, and gaps were treated as missing data. Trees were inferred using the heuristic search option with TBR branch swapping and 1,000 random sequence additions. Max-trees were set to 5,000, branches of zero length were collapsed, and all parsimonious trees were saved. Clade robustness was assessed using bootstrap (BT) analysis with 1,000 replicates. Descriptive tree statistics—tree length (TL), consistency index (CI), retention index (RI), rescaled consistency index (RC), and homoplasy index (HI)—were calculated for each maximum parsimonious tree generated. The combined dataset was also analyzed using ML in RAxML-HPC2 through the Cipres Science Gateway. Branch support (BS) for ML analysis was determined by 1,000 bootstrap replicates (Wang et al. 2024; Yang et al. 2025a).

The best evolutionary model of each alignment was estimated using jModelTest (Guindon and Gascuel 2003; Posada 2008) under the Akaike information criterion. MrModeltest 2.3 (Nylander 2004) was used to determine the best-fit evolution model for the dataset for Bayesian inference (BI). BI was calculated with MrBayes version 3.2.7a (Ronquist et al. 2012). Four Markov chains were run for 2 runs from random starting trees for 1.45 million generations for ITS+nLSU+RPB1+RPB2+TEF1 (Fig. 1). Four Markov chains were run for 2 runs from random starting trees for 500 thousand generations for ITS+nLSU+RPB1+RPB2+TEF1 (Fig. 2). Four Markov chains were run for 2 runs from random starting trees for 2.42 million generations for ITS+nLSU+RPB1+RPB2+TEF1 (Fig. 3). Four Markov chains were run for 2 runs from random starting trees for 500 thousand generations for ITS+nLSU+RPB2+TEF1 (Fig. 4). The first one-fourth of all generations was discarded as burn-in. The majority rule consensus tree of all remaining trees was calculated. Branches were considered as significantly supported if they received a maximum likelihood bootstrap value (BS) > 70%, a maximum parsimony bootstrap value (BT) > 50%, or Bayesian posterior probabilities (BPP) > 0.95.

## ﻿Results

### ﻿Molecular phylogeny

In Irpicaceae analyses, the ITS+nLSU+RPB1+RPB2+TEF1 dataset (Fig. 1) included sequences from 69 fungal specimens representing 38 taxa. The dataset had an aligned length of 5047 characters, of which 3162 characters are constant, 492 are variable and parsimony-uninformative, and 1393 are parsimony-informative. Maximum parsimony analysis yielded 4 equally parsimonious trees (TL = 1226, CI = 0.3100, HI = 0.6900, RI = 0.5925, and RC = 0.1836). The best model for the ITS+nLSU+RPB1+RPB2+TEF1 dataset estimated and applied in the Bayesian analysis was GTR+I+G. Bayesian analysis and ML analysis resulted in a similar topology as in the MP analysis, with an average standard deviation of split frequencies = 0.009515 (BI), and the effective sample size (ESS) for Bayesian analysis across the two runs is double the average ESS (avg ESS = 640).

In *Efibula* analyses, the ITS+nLSU+RPB1+RPB2+TEF1 dataset (Fig. 2) included sequences from 42 fungal specimens representing 18 taxa. The dataset had an aligned length of 4694 characters, of which 3227 characters are constant, 225 are variable and parsimony-uninformative, and 1242 are parsimony-informative. Maximum parsimony analysis yielded 2 equally parsimonious trees (TL = 409, CI = 0.5281, HI = 0.4719, RI = 0.8381, and RC = 0.4426). The best model for the ITS+nLSU+RPB1+RPB2+TEF1 dataset estimated and applied in the Bayesian analysis was GTR+I+G. Bayesian analysis and ML analysis resulted in a similar topology as in the MP analysis, with an average standard deviation of split frequencies = 0.009147 (BI), and the effective sample size (ESS) for Bayesian analysis across the two runs is double the average ESS (avg ESS = 280).

In Irpicaceae analyses, the ITS+nLSU+RPB1+RPB2+TEF1 dataset (Fig. 3) included sequences from 75 fungal specimens representing 50 taxa. The dataset had an aligned length of 4958 characters, of which 2918 characters are constant, 659 are variable and parsimony-uninformative, and 1381 are parsimony-informative. Maximum parsimony analysis yielded 4 equally parsimonious trees (TL = 6725, CI = 0.4736, HI = 0.5264, RI = 0.5843, and RC = 0.2767). The best model for the ITS+nLSU+RPB1+RPB2+TEF1 dataset estimated and applied in the Bayesian analysis was GTR+I+G. Bayesian analysis and ML analysis resulted in a similar topology as in the MP analysis, with an average standard deviation of split frequencies = 0.009989 (BI), and the effective sample size (ESS) for Bayesian analysis across the two runs is double the average ESS (avg ESS = 1010).

In *Scopuloides* analyses, the ITS+nLSU+RPB2+TEF1 dataset (Fig. 4) included sequences from 20 fungal specimens representing 10 taxa. The dataset had an aligned length of 3412 characters, of which 2813 characters are constant, 351 are variable and parsimony-uninformative, and 248 are parsimony-informative. Maximum parsimony analysis yielded 13 equally parsimonious trees (TL = 314, CI = 0.6465, HI = 0.3535, RI = 0.7147, and RC = 0.4620). The best model for the ITS+nLSU+RPB2+TEF1 dataset estimated and applied in the Bayesian analysis was GTR+I+G. Bayesian analysis and ML analysis resulted in a similar topology as in the MP analysis, with an average standard deviation of split frequencies = 0.009164 (BI), and the effective sample size (ESS) for Bayesian analysis across the two runs is double the average ESS (avg ESS = 454).

Based on the ITS+nLSU+RPB1+RPB2+TEF1 dataset (Fig. 1) of the family Irpicaceae, *Crystallicutisalbomarginata* was sister to *C.serpens*. The ITS+nLSU+RPB1+RPB2+TEF1 dataset (Fig. 2) phylogenetic analysis of the genus *Efibula* showed that the two species were confirmed to be grouped together and clustered into the genus *Efibula*, in which *E.punctata* was sister to *E.intertexta* (Sheng H. Wu) C.C. Chen & Sheng H. Wu; *Efibulaglossophora* is closely related to *E.intertexta* and *E.hainanensis* Yue Li & S.H. He. The phylogenetic analysis based on the ITS+nLSU+RPB1+RPB2+TEF1 dataset (Fig. 3) supported *Scopuloidesfarinacea* as a member of Meruliaceae, and based on the ITS+nLSU+RPB2+TEF1 dataset (Fig. 4) phylogenetic analysis, it was shown that the new species *S. farinacea* was sister to *S. allantoidea* C.C. Chen & Sheng H. Wu.

### ﻿Taxonomy

#### ﻿ Crystallicutis albomarginata

Taxon classificationFungiPolyporalesIrpicaceae

Z.R. Gu & C.L. Zhao sp. nov.

A3E60E9C-C47F-5A36-8CCC-BB1B4DF86429

858807

Figs 5 , 6 , 7

##### Diagnosis.

Differs from other *Crystallicutis* species by its slightly pink to orange basidiomata when dry, a monomitic hyphal system with simple septa, and narrowly ellipsoid basidiospores measuring 3.7–4.4 × 1.9–2.8 µm.

**Figure 5.**
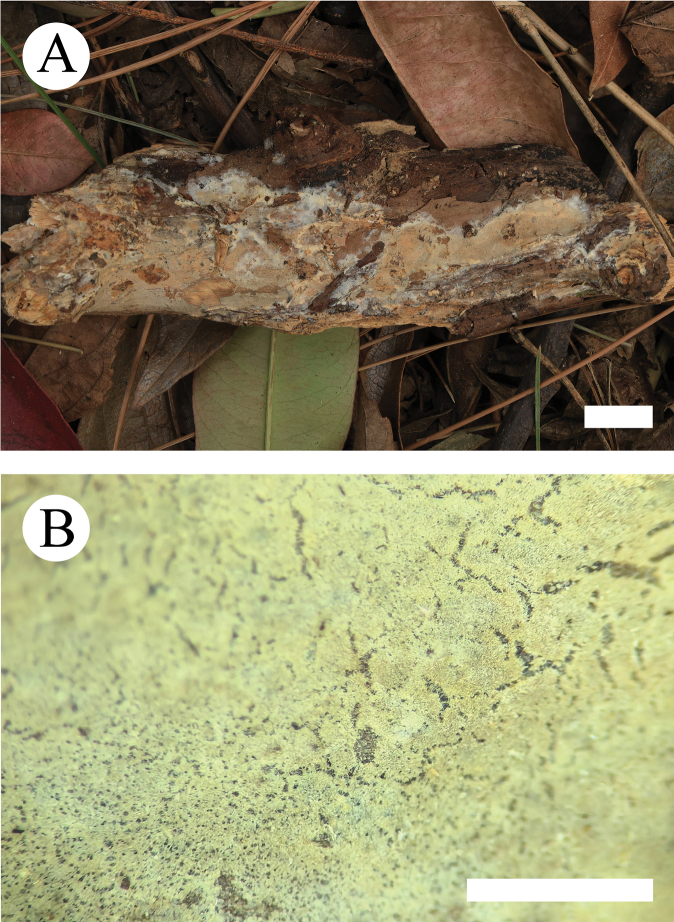
Basidiomata of *Crystallicutisalbomarginata* (holotype, CLZhao 31506). **A.** Basidiomata on the substrate; **B.** Macroscopic characteristics of hymenophore. Scale bars: 1 cm (**A**); 1 mm (**B**).

##### Holotype.

China • Yunnan Province, Zhaotong, Wumengshan National Nature Reserve, GPS coordinates: 22°77'N, 104°29'E, altitude 1800 m asl., on the fallen angiosperm branch, leg. C.L. Zhao, 25 August 2023, CLZhao 31506, GenBank: ITS = PV470539, nLSU = PV474184 (SWFC).

**Figure 6.**
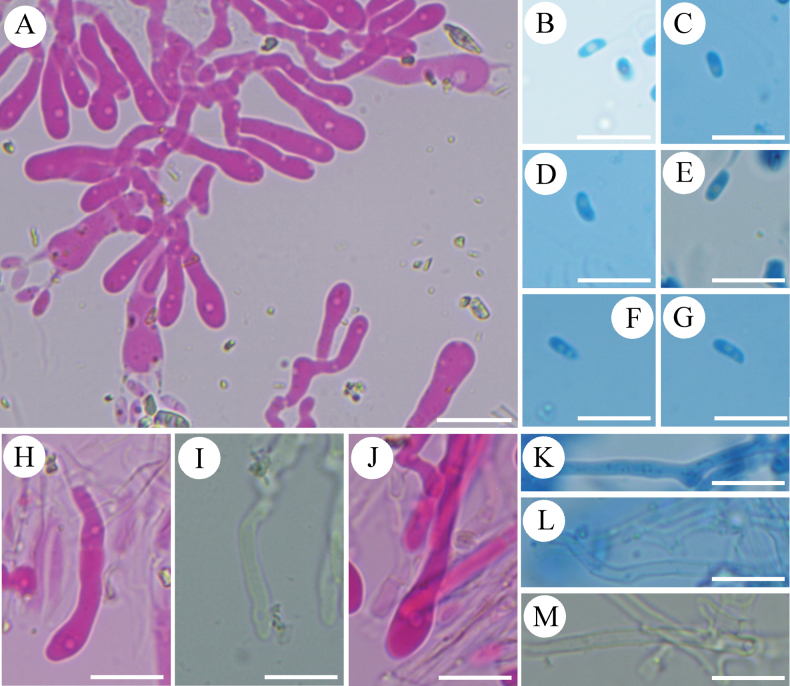
Sections of hymenium of *Crystallicutisalbomarginata* (holotype, CLZhao 31506). **A.** Basidia and basidioles; **B–G.** Basidiospores; **H–J.** Cystidia; **K–M.** Part of the generative hyphae. Scale bars: 10 µm (**A**–**M**); 10 × 100 Oil.

##### Etymology.

*Albomarginata* (Latin or Greek origin): refers to the white margin of the basidiomata.

**Figure 7.**
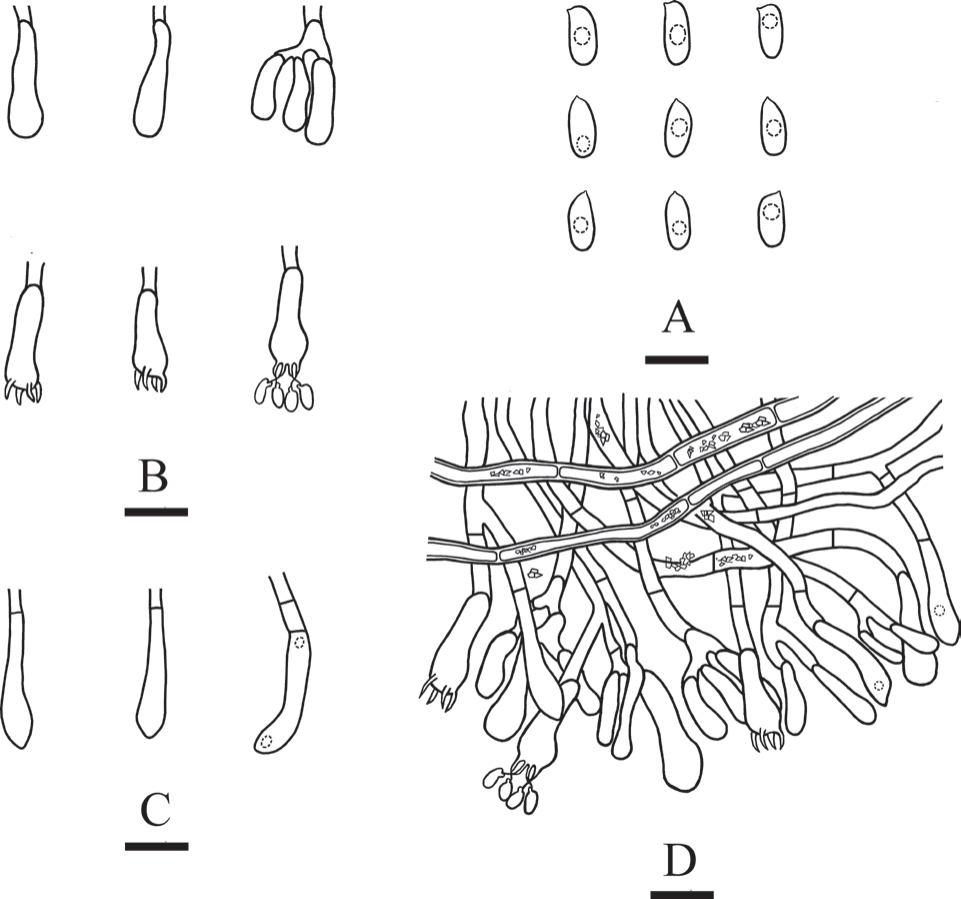
Microscopic structures of *Crystallicutisalbomarginata* (holotype, CLZhao 31506). **A.** Basidiospores; **B.** Basidia and basidioles; **C.** Cystidia; **D.** Part of the vertical section of hymenium. Scale bars: 5 µm (**A**); 10 µm (**B–D**).

##### Description.

***Basidiomata*.** Annual, resupinate, adnate, membranaceous, without odor or taste when fresh, up to 10 cm long, 2.5 cm wide, and 200 μm thick at center. Hymenial surface smooth, cream to pale pink when fresh, turning to slightly pink to orange upon drying. Sterile margin narrow, cream to pale pink, up to 1 mm.

***Hyphal system*.** Monomitic; generative hyphae with simple septa, colorless, thin- to slightly thick-walled, rarely branched, interwoven, 2.1–3 µm in diameter, IKI–, CB–; tissues unchanged in KOH. ***Hymenium*.** Cystidia fusiform, colorless, thin-walled, smooth, 12.5–27 × 3.5–6 µm. Basidia clavate, thin-walled, smooth, slightly flexuous, with four sterigmata and a simple septum at the base, 12.1–19.4 × 3.8–6 µm; basidioles in shape similar to basidia, but slightly smaller; subhymenial hyphae covered with smaller irregularly shaped colorless crystals. ***Basidiospores*.** Basidiospores narrowly ellipsoid, colorless, thin-walled, smooth, with 1-2 guttules, IKI–, CB–, (3.5–)3.7–4.4(–4.6) × (1.6–)1.9–2.8 µm, L = 4.06 µm, W = 2.33 µm, Q = 1.58–1.95 (n = 60/2).

##### Additional specimen examined (paratype).

China • Yunnan Province, Zhaotong, Wumengshan National Nature Reserve, GPS coordinates: 22°77'N, 104°29'E, altitude 1800 m asl., on the fallen angiosperm branch, leg. C.L. Zhao, 25 August 2023, CLZhao 31409, GenBank: ITS = PV470538, nLSU = PV474183 (SWFC).

#### ﻿ Efibula glossophora

Taxon classificationFungiPolyporalesIrpicaceae

Z.R Gu & C.L. Zhao sp. nov.

A8A8A946-CA11-5FE4-BD89-7965FB082FF4

858810

Figs 8 , 9 , 10

##### Diagnosis.

Differs from other *Efibula* species by its hard, membranous, slightly yellow to yellow basidiomata; a monomitic hyphal system with simple-septa generative hyphae; and ellipsoid basidiospores measuring 3.8–6 × 2.6–3.7 µm.

**Figure 8.**
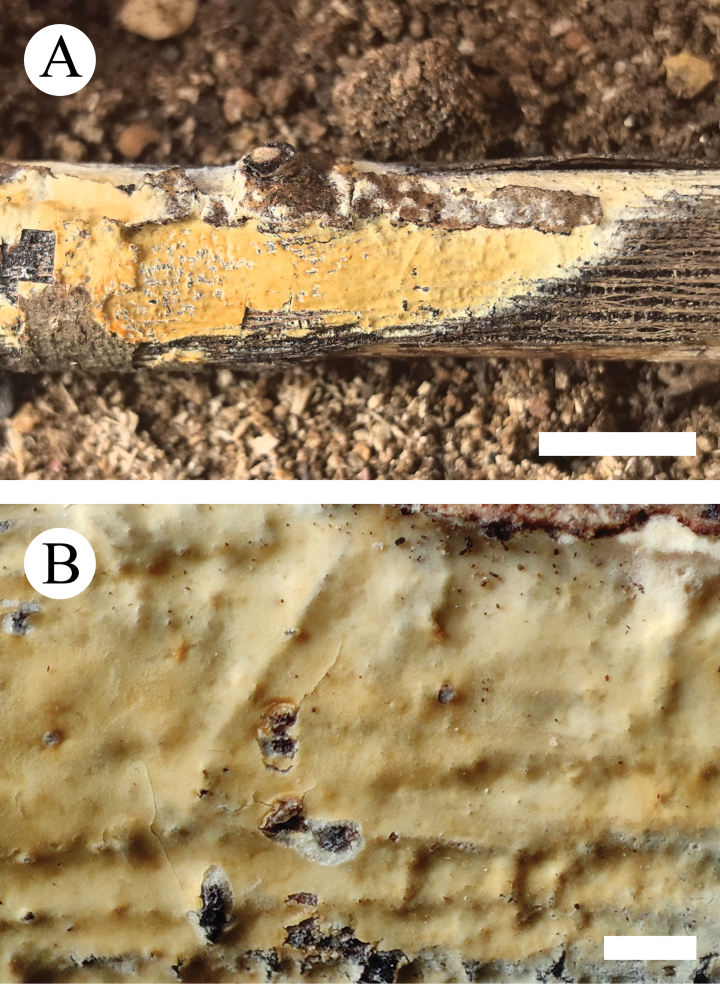
Basidiomata of *Efibulaglossophora* (holotype, CLZhao 22744). **A.** Basidiomata on the substrate; **B.** Macroscopic characteristics of hymenophore. Scale bars: 1 cm (**A**); 1 mm (**B**).

**Figure 9.**
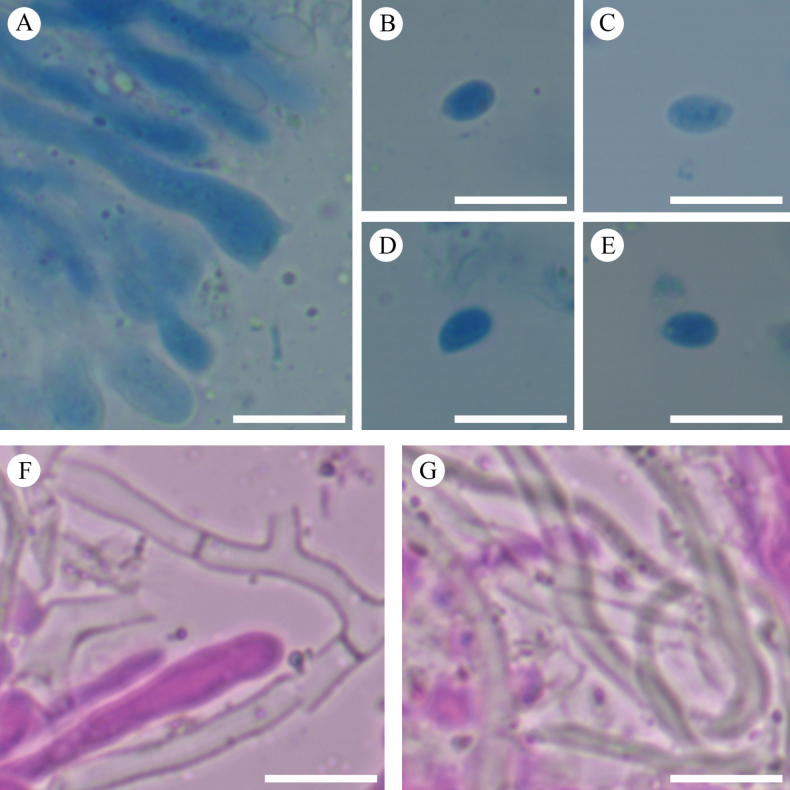
Sections of hymenium of *Efibulaglossophora* (holotype, CLZhao 22744). **A.** Basidia and basidioles; **B–E.** Basidiospores; **F, G.** Part of the generative hyphae. Scale bars: 10 µm (**A**–**G**); 10 × 100 Oil.

##### Holotype.

China • Yunnan Province, Dali, Weishan County, Leqiu Town, Zhongyao Village, GPS coordinates: 25°01'N, 100°4'E, altitude 1900 m asl., on the fallen angiosperm branch, leg. C.L. Zhao, 19 July 2022, CLZhao 22744, GenBank: ITS = PV470540, nLSU = PV474185 (SWFC).

**Figure 10.**
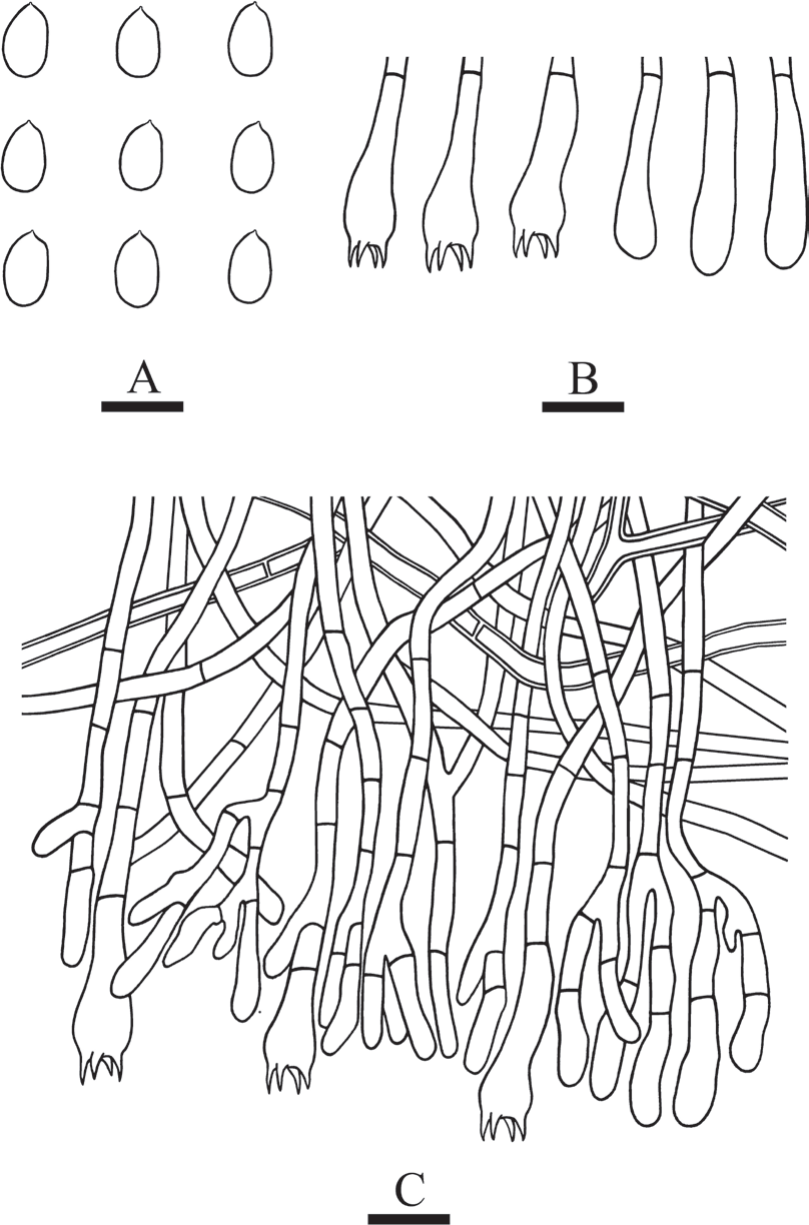
Microscopic structures of *Efibulaglossophora* (holotype, CLZhao 22744). **A.** Basidiospores; **B.** Basidia and basidioles; **C.** Part of the vertical section of hymenium. Scale bars: 5 µm (**A**); 10 µm (**B**, **C**).

##### Etymology.

*Glossophora* (Latin or Greek origin): refers to the smooth hymenial surface of the type specimen.

##### Description.

***Basidiomata*.** Annual, resupinate, closely adnate, hard membranaceous, without odor or taste when fresh, up to 9 cm long, 2.5 cm wide, and 300 μm thick at center. Hymenophore smooth, buff to slightly yellow when fresh, slightly yellow to yellow upon drying. Sterile margin narrow, cream to slightly yellow, up to 1 mm.

***Hyphal system*.** Monomitic; generative hyphae with simple septa, colorless, thin- to slightly thick-walled, smooth, rarely branched, interwoven, 3–3.5 µm in diameter, IKI–, CB–; tissues unchanged in KOH. ***Hymenium*.** Cystidia and cystidioles absent. Basidia long clavate, slightly flexuous, with four sterigmata and a simple septum at the base, 21.5–26.7 × 5.7–7.4 µm; basidioles numerous, in shape similar to basidia but smaller. ***Basidiospores*.** Basidiospores ellipsoid, colorless, thin-walled, smooth, IKI–, CB–, (3.6–)3.8–6(–7.5) × (2–)2.6–3.7(–4.3) µm, L = 5.3 µm, W = 3.3 µm, Q = 1.63 (n = 30/1).

#### ﻿ Efibula punctata

Taxon classificationFungiPolyporalesIrpicaceae

Z.R Gu & C.L. Zhao sp. nov.

828A0354-AC55-5182-AA40-FD0E8B593EA3

858811

Figs 11 , 12 , 13

##### Diagnosis.

Differs from other *Efibula* species by its membranaceous, slightly gray to pale brown basidiomata, a monomitic hyphal system with simple-septate generative hyphae, and ellipsoid basidiospores measuring 4.3–6.2 × 2.2–3.3 µm.

**Figure 11.**
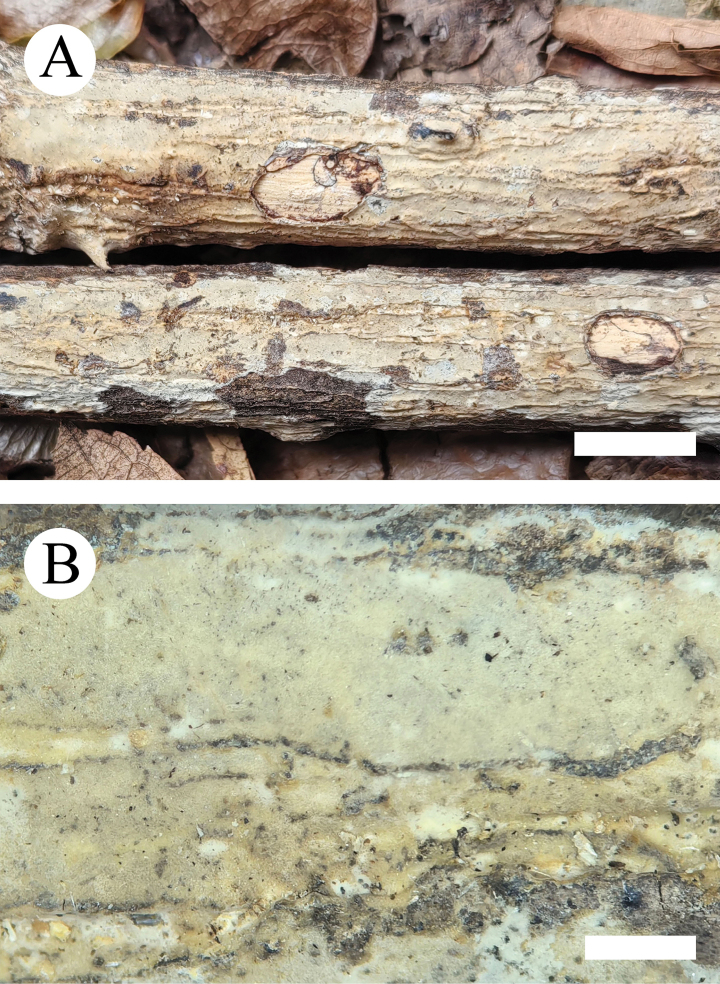
Basidiomata of *Efibulapunctata* (holotype, CLZhao 30011). **A.** Basidiomata on the substrate; **B.** Macroscopic characteristics of hymenophore. Scale bars: 1 cm (**A**); 1 mm (**B**).

##### Holotype.

China • Yunnan Province, Dehong, Yingjiang County, Tongbiguan Provincial Nature Reserve, GPS coordinates: 27°77'N, 104°25'E, altitude 1900 m asl., on the fallen angiosperm branch, leg. C.L. Zhao, 18 July 2023, CLZhao 30011, GenBank: ITS = PV470544, nLSU = PV474189 (SWFC).

**Figure 12.**
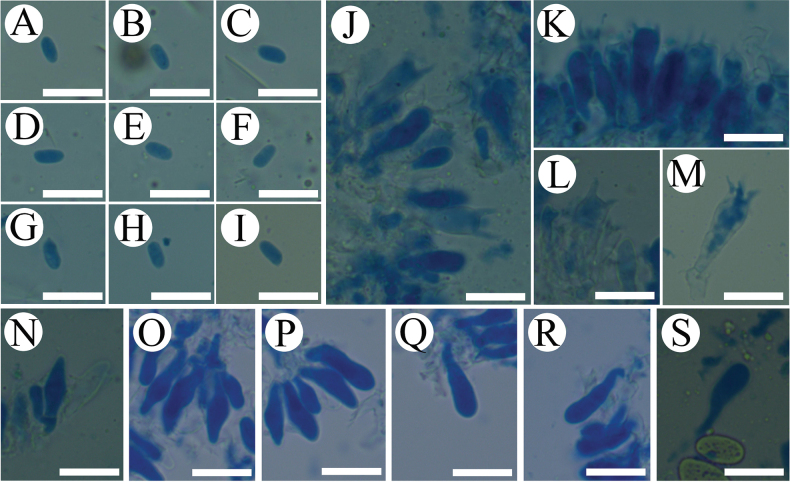
Sections of hymenium of *Efibulapunctata* (holotype, CLZhao 30011). **A–I.** Basidiospores; **J–M.** Basidia; **N–P.** Cystidoles; **Q–S.** Basidioles. Scale bars: 10 µm (**A–S**); 10 × 100 Oil.

##### Etymology.

*Punctata* (Latin or Greek origin): refers to the species having cushion-shaped hymenial surface of the type specimens.

**Figure 13.**
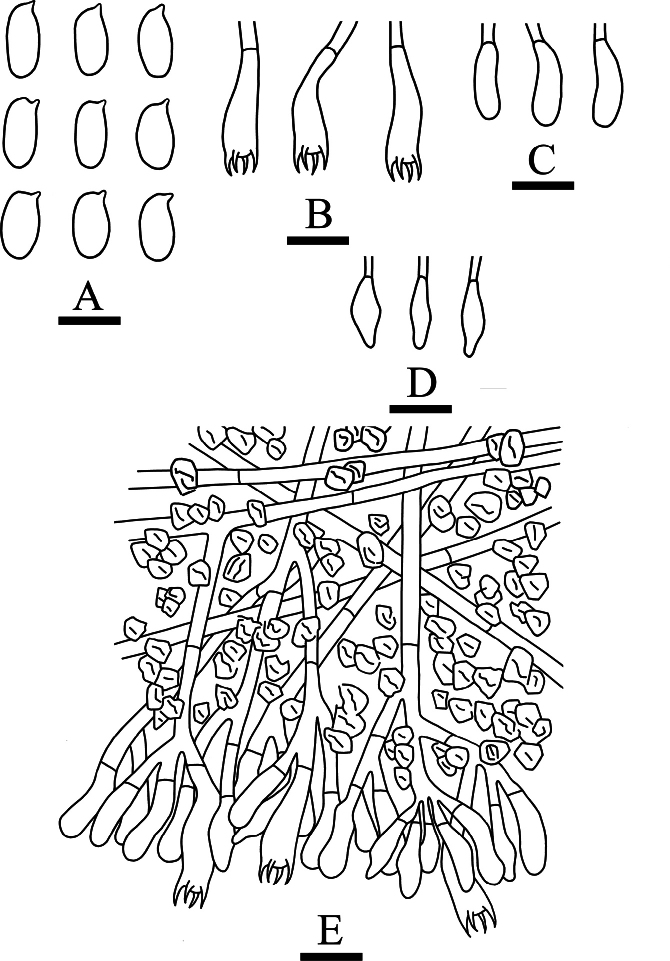
Microscopic structures of *Efibulapunctata* (holotype, CLZhao 30011). **A.** Basidiospores; **B.** Basidia; **C.** Basidioles; **D.** Cystidoles. Scale bars: 5 µm (**A**); 10 µm (**B–D**).

##### Description.

***Basidiomata*.** Annual, resupinate, closely adnate, thin membranaceous, without odor or taste when fresh, up to 10 cm long, 2 cm wide, and 200 μm thick at center. Hymenophore smooth, cream to slightly brown when fresh, slightly gray to pale brown upon drying. Sterile margin narrow, cream to slightly brown, up to 1 mm.

***Hyphal system*.** Monomitic; generative hyphae with simple septa, colorless, thin-walled, smooth, branched, interwoven, 1.5–3.5 µm in diameter; IKI–, CB–; tissues unchanged in KOH. ***Hymenium*.** Cystidia absent, cystidoles mostly subfusiform, colorless, thin-walled, smooth, 9.8–22 × 2.8–5.7 µm. Basidia subcylindrical to subclavate, slightly flexuous, with a basal simple septum and four sterigmata, 11.8–19.4 × 3.7–5.9 µm; basidioles numerous, in shape similar to basidia but smaller. ***Basidiospores*.** Basidiospores ellipsoid, colorless, thin-walled, IKI–, CB–, (3.6–)4.3–6.2(–6.7) × (1.8–)2.2–3.3(–3.9) µm, L = 5.17 µm, W = 2.74 µm, Q = 1.81–1.92 (n = 240/8).

##### Additional specimens examined (paratypes).

China • Yunnan Province, Dali, Weishan County, Leqiu Town, Zhongyao Village, GPS coordinates: 25°01'N, 100°4'E, altitude 1900 m asl., on the fallen angiosperm branch, leg. C.L. Zhao, 19 July 2022, CLZhao 22764, GenBank: ITS = PV470541, nLSU = PV474186 (SWFC). • Dehong, Yingjiang County, Tongbiguan Provincial Nature Reserve, GPS coordinates: 24°42'N, 97°56'E, altitude 850 m asl., on the fallen angiosperm branch, leg. C.L. Zhao, 17 July 2023, CLZhao 29674, GenBank: ITS = PV470542; nLSU = PV474187; CLZhao 29678, GenBank: ITS = PV470543; nLSU = PV474188; on the fallen angiosperm branch, leg. C.L. Zhao, 18 July 2023, CLZhao 30054, GenBank: ITS = PV470545; nLSU = PV474190; on the fallen angiosperm branch, leg. C.L. Zhao, 20 July 2023, CLZhao 30615, GenBank: ITS = PV470546; nLSU = PV474191, CLZhao 30637, GenBank: ITS = PV470547; nLSU = PV474192, CLZhao 30648, GenBank: ITS = PV470548; nLSU = PV474193, CLZhao 30659, GenBank: ITS = PV470549; nLSU = PV474194, CLZhao 30664, GenBank: ITS = PV470550; nLSU = PV474195, CLZhao 30691, GenBank: ITS = PV470551; nLSU = PV474196, CLZhao 30718, GenBank: ITS = PV470552; nLSU = PV474197 (SWFC).

#### ﻿ Scopuloides farinacea

Taxon classificationFungiPolyporalesMeruliaceae

Z.R Gu & C.L. Zhao sp. nov.

057CCA15-2797-5C3C-925C-AAC27FB19EB4

858812

Figs 14 , 15 , 16

##### Diagnosis.

Differs from other *Scopuloides* species by its coriaceous, pale cream to buff basidiomata, a monomitic hyphal system with simple-septate generative hyphae, and ellipsoid basidiospores measuring 2.8–3.5 × 1.4–2 µm.

**Figure 14.**
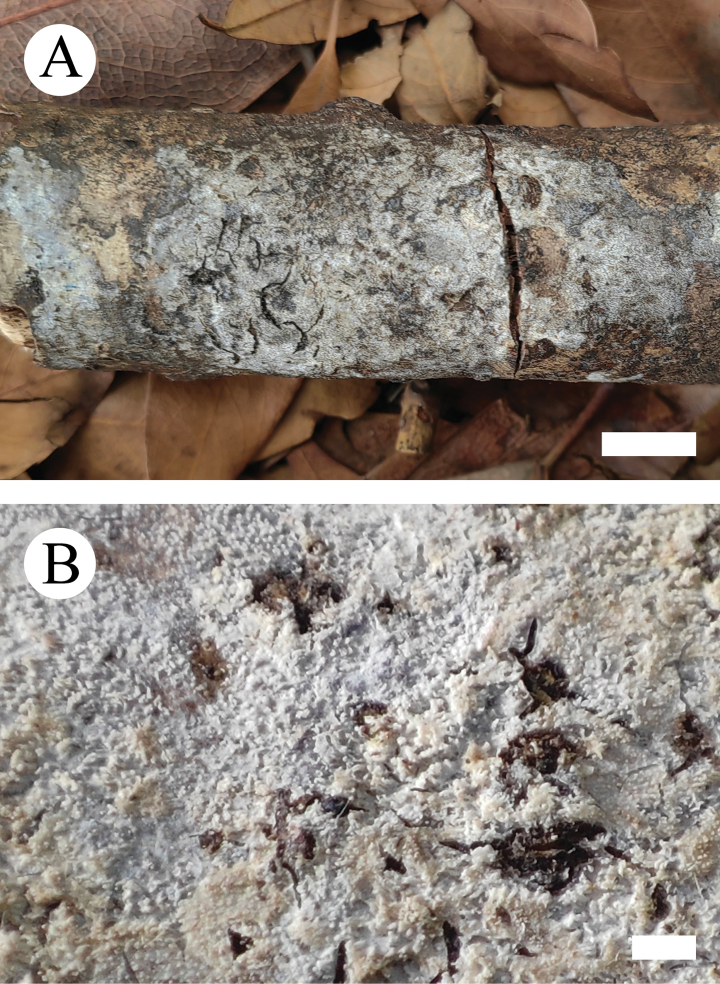
Basidiomata of *Scopuloidesfarinacea* (holotype, CLZhao 30181). **A.** Basidiomata on the substrate; **B.** Macroscopic characteristics of hymenophore. Scale bars: 1 cm (**A**); 1 mm (**B**).

##### Holotype.

China • Yunnan Province, Dehong, Yingjiang County, Tongbiguan Provincial Nature Reserve, GPS coordinates: 24°42'N, 97°56'E, altitude 850 m asl., on the fallen angiosperm branch, leg. C.L. Zhao, 19 July 2023, CLZhao 30181, GenBank: ITS = PV470559; nLSU = PV474204 (SWFC).

**Figure 15.**
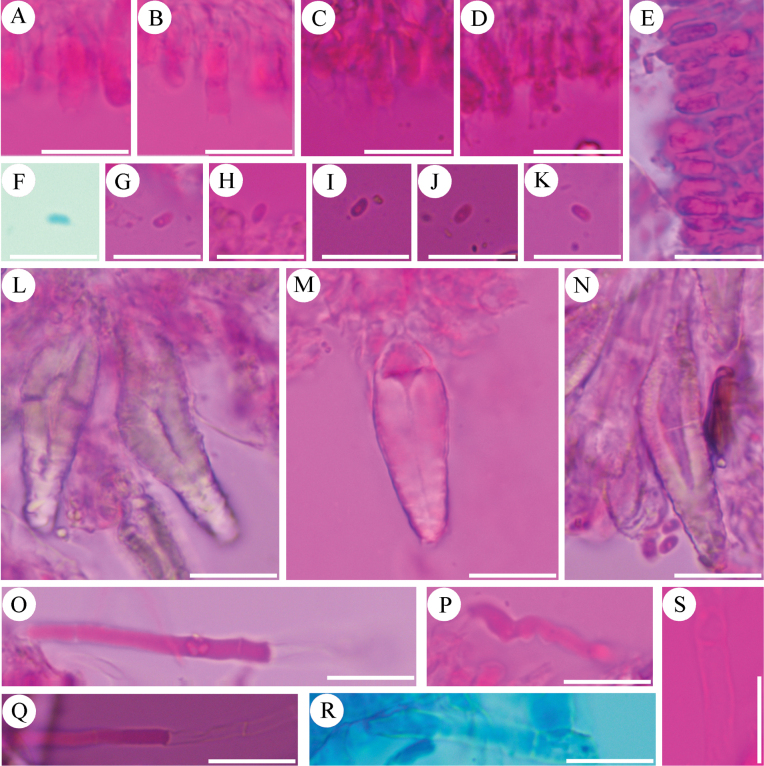
Sections of hymenium of *Scopuloidesfarinacea* (holotype, CLZhao 30181). **A–E.** Basidia and basidioles; **F–K.** Basidiospores; **L–N.** Cystidia; **O–S.** Part of the generative hyphae. Scale bars: 10 µm (**A**–**S**); 10 × 100 Oil.

##### Etymology.

*Farinacea* (Latin or Greek origin): refers to the farinaceous hymenial surface of the type specimens.

**Figure 16.**
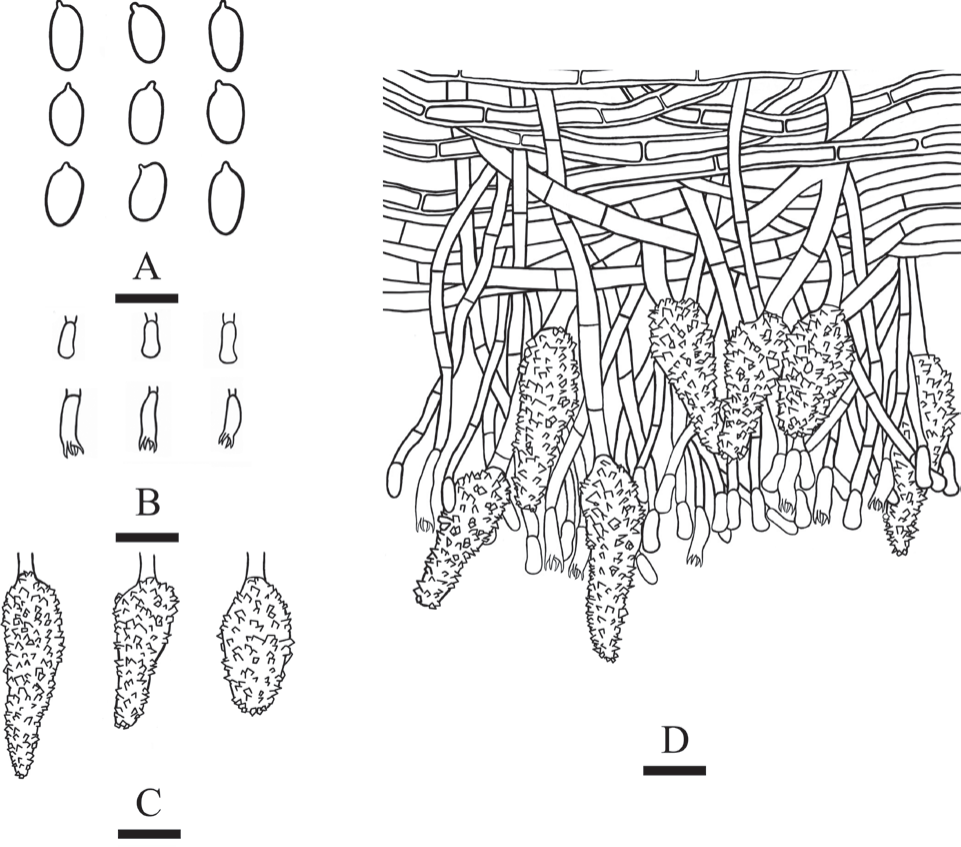
Microscopic structures of *Scopuloidesfarinacea* (holotype, CLZhao 30181). **A.** Basidiospores; **B.** Basidia and basidioles; **C.** Cystidia; **D.** Part of the vertical section of hymenium. Scale bars: 5 µm (**A**); 10 µm (**B–D**).

##### Description.

***Basidiomata*.** Annual, resupinate, closely adnate, coriaceous, without odor or taste when fresh, up to 20 cm long, 3 cm wide, and 400 μm thick at center. Hymenophore grandinoid, white to cream when fresh, pale cream to buff upon drying. Sterile margin white to pale cream, up to 1 mm.

***Hyphal system*.** Monomitic; generative hyphae with simple septa, colorless, thin- to slightly thick-walled, smooth, branched, more or less parallel with substrate, 1.7–5.5 µm in diameter, IKI–, CB–; tissues unchanged in KOH. ***Hymenium*.** Lamprocystidia abundant, conical or subulate, colorless, simple-septate at base, heavily encrusted, originating from trama or subiculum, immersed or projecting, 21.2–39.5 × 6–13 µm. Basidia subcylindrical, slightly flexuous, with a basal simple septum and four sterigmata, 4.6–8 × 1.8–2.7 µm; basidioles numerous, in shape similar to basidia but slightly smaller. ***Basidiospores*.** Basidiospores ellipsoid, colorless, thin-walled, smooth, IKI–, CB–, (2.6–)2.8–3.5(–3.7) × (1.2–)1.4–2 µm, L = 3.08 µm, W = 1.65 µm, Q = 1.79–1.92 (n = 90/3).

##### Additional specimens examined (paratypes).

China • Yunnan Province, Zhaotong, Yiliang County, Xiaocaoba Town, Wumengshan National Nature Reserve, Wangfu Waterfall, GPS coordinates: 22°19'N, 103°43'E, altitude 1800 m asl., on the fallen angiosperm branch, leg. H.M. Zhou, 18 August 2024, HMZhou 153, GenBank: ITS = PV470560, nLSU = PV474205; • Dehong, Yingjiang County, Tongbiguan Provincial Nature Reserve, GPS coordinates: 24°42'N, 97°56'E, altitude 850 m asl., on the angiosperm trunk, leg. C.L. Zhao, 18 July 2023, CLZhao 30005, GenBank: ITS = PV470558; nLSU = PV474203 (SWFC).

## ﻿Discussion

In the present study, four new species, *Crystallicutisalbomarginata*, *Efibulaglossophora*, *E.punctata*, and *Scopuloidesfarinacea*, are described based on phylogenetic analyses and morphological characteristics.

Phylogenetically, three new taxa were grouped into the family Irpicaceae based on the ITS+nLSU+RPB1+RPB2+TEF1 dataset, in which *Crystallicutisalbomarginata* was sister to *C.serpens* (Fig. 1). Morphologically, *C.serpens* has bigger basidia (21–32 × 4.5–5 μm; El-Gharabawy et al. 2021). *Efibulapunctata* and *E.glossophora* were grouped with *E.yunnanensis* C.L. Zhao, *E.gracilis* Floudas & Hibbett, *E.hainanensis*, and *E.clarkii* Floudas & Hibbett (Fig. 1). The observed topology is strongly supported and consistent with previous findings (El-Gharabawy et al. 2021). Phylogenetic analysis based on the ITS+nLSU+RPB1+RPB2+TEF1 dataset showed that the two species were confirmed to be grouped together and clustered into the genus *Efibula*, consistent with previous research (Dong et al. 2024). *Efibulaglossophora* is closely related to *E.intertexta* and *E.hainanensis* (Fig. 2); *E.punctata* was sister to *E.intertexta* (Fig. 2). Morphologically, *Efibulaglossophora* is similar to *E.intertexta* and *E.hainanensis*, but *E.intertexta* has bigger basidia (30–35 × 4.5–5 μm; Chen et al. 2021), and *E.hainanensis* has rare cystidia (Li et al. 2022). *Efibulapunctata* differs from *E.intertexta* by having bigger basidia (30–35 × 4.5–5 μm; Chen et al. 2021).

The morphological distinctions among *Crystallicutis* species, including the newly described taxon *Crystallicutisalbomarginata*, are delineated through the comparative analysis presented in Table 2. Comparative characteristics of *Efibula* species are tabulated in Table 3 to establish taxonomic differentiation for the newly proposed species.

**Table 2.** A morphological comparison between *Crystallicutisalbomarginata* and four similar species in the genus *Crystallicutis*.

Species name	Hymenial surface	Generative Hyphae	Cystidia	Basidia	Basidiospores	References
***Crystallicutisalbomarginata***	**Membranaceous, smooth, slightly pink to orange**	**Monomitic, simple septa, thin to slightly thick-walled, rarely branched**	**Fusiform, thin-walled, 12.5–20 × 3.5–6 µm**	**Clavate, 12.1–19.4 × 3.8–6 µm**	**Narrowly ellipsoid, thin-walled, 1-2 guttulae, 3.7–4.4 × 1.9–2.8 µm**	**Present study**
*Crystallicutisdamiettensis*	Ceraceous, tuberculate to papillate-warty, honey-yellow	Monomitic, clamp, frequent stumpy branches	Long, spear-shaped, thin-walled, 22–25 × 4.0–5.0 μm; Cystidioles are fusiform, 18–22 × 3.0–4.0 μm	Clavate, 12.0–15.0 × 6.0–7.5 μm	Ovoid to ellipsoid, thick-walled, 4.0–5.0 × 3.0–3.5 μm	El-Gharabawy et al. (2016)
*Crystallicutishuangshanensis*	Honey-yellow to olivaceous-buff	Monomitic, simple septa, thin- to slightly thick-walled, moderately branched	—	Clavate, 13.4–19.5 × 3.6–5.2 µm	Broadly ellipsoid; thin-walled, 3–3.9 × 2–2.4 µm	El-Gharabawy et al. (2021
*Crystallicutisrajchenbergii*	Non-ceraceous, smooth with small cracks, honey-yellow colored with tan to brownish	Monomitic, thin-walled, densely branched	Clavate with a sharp apex,thick walled, 25–30 × 4–5 μm; Cystidioles fusiform, thick-walled, 10 × 3 μm	Ovoid to clavate, 8–10 × 5–6 μm	Subellipsoid, thick-walled, 10–15 × 3–6 μm	El-Gharabawy et al. (2016)
*Crystallicutisserpens*	Ceraceous, smooth to slightly merulioid, hymenial surface buff, rosy buff to salmon	Monomitic, clamp, thin-walled	—	Narrowly clavate, 21–32 × 4.5–5 µm	Narrowly ellipsoid, thin-walled, 3.5–5 × 1.5–2.5 µm	Maekawa (1994)

**Table 3.** A morphological comparison between two new species and similar species in the genus *Efibula*.

Species name	Hymenial surface	Cystidia	Basidia (μm)	Basidiospores (μm)	References
*E.americana*	Smooth to reticulate	Absent	20–32 × 5–8	Ellipsoid to cylindrical; 5.3–6.5 × 3–3.8	Floudas and Hibbett (2015)
*E.clarkii*	Slightly tuberculate	Absent	25–39 × 5–7.5	Oblong to ellipsoid; 6–7 × 3–3.5	Floudas and Hibbett (2015)
***E.glossophora***	**Smooth**	**Absent**	**21.5–26.7 × 5.7–7.4**	**Ellipsoid; 3.8–6 × 2.6–3.7**	**Present study**
*E.gracilis*	Smooth	Absent	17–30 × 5–6.5	Ellipsoid to oblong; 5.5–7 × 3.3–4	Floudas and Hibbett (2015)
*E.grandinosa*	Grandinioid	Absent	36–43 × 5–7	Ellipsoid; 6–6.8 × 3.7–4	Li et al. (2022)
*E.hainanensis*	Smooth	Rare	15–26 × 4–6	Ellipsoid to broadly ellipsoid; 4.2–5.5 × 2.8–3.2	Li et al. (2022)
*E.intertexta*	Smooth	Absent	30–35 × 4.5–5	Cylindrical; 5.6–6.4 × 2.2–2.6	Chen et al. (2021)
*E.matsuensis*	Smooth	Absent	18–25 × 6.5–8	Ellipsoid to cylindrical; 7.4–8.6 × 3.8–4.4	Chen et al. (2021)
***E.punctata***	**Smooth**	**Absent**	**11.8–19.4 × 3.7–5.9**	**Ellipsoid; 4.3–6.2 × 2.2–3.3**	**Present study**
*E.rodriguezarmasiae*	Smooth to tuberculate	Absent	35–48 × 6–8	Ellipsoid; 6–7 × 4–5	Boonmee et al. (2021)
*E.shenghuae*	Smooth to grandinioid	Absent	23–38 × 4.5–7	Oblong ellipsoid; 6–6.5 × 3–3.5	Li et al. (2022)
*E.subglobispora*	Smooth	Absent	30–40 × 6.5–8	Broadly ellipsoid to subglobose; 6.4–8.1 × 4.5–5.8	Chen et al. (2021)
*E.taiwanensis*	Smooth	Absent	24–44 × 6–8	Broadly elliosoid to ovoid; 5.8–6.5 × 4–4.5	Li et al. (2022)
*E.tropica*	Smooth	Absent	20–40 × 5.5–8	Broadly elliosoid; 6.4–7.7 × 3.7–4.4	Chen et al. (2021)
*E.tuberculata*	Smooth to slightly tuberculate	Absent	18–35 × 5–6	Ellipsoid; 5.3–6.4 × 3.4–4.3	Chen et al. (2021)
*E.turgida*	Smooth	Absent	26–30 × 6.5–7	Cylindrical; 6.6–8.2 × 3.3–3.9	Chen et al. (2021)
*E.yunnanensis*	Mainly Smooth, sometimes slightly tuberculate	Absent	27–38 × 6–7	Broadly ellipsoid; 6.6–8 × 3.9–4.7	Chen et al. (2021)

Phylogenetically, four *Scopuloides* species—*S. allantoidea*, *S. dimorpha* (Sang H. Lin & Z.C. Chen) C.C. Chen & Sheng H. Wu, *S. hydnoides*, and *S. rimosa* (Cooke) Jülich—grouped together and formed a strongly supported clade based on the ITS+nLSU+TEF1+mt-SSU+GAPDH+RPB1+RPB2 sequences (Chen et al. 2021; Zhao et al. 2023). In our study, phylogenetic analysis based on ITS+nLSU+RPB1+RPB2+TEF1 data provided molecular evidence supporting *Scopuloidesfarinacea* as a member of Meruliaceae, in which *S. farinacea* was confirmed to group with *S. allantoidea* and *S. rimosa* (Fig. 3), consistent with previous findings (Liu et al. 2022; Zhou et al. 2024; Li et al. 2025). Based on ITS+nLSU+RPB2+TEF1 phylogenetic analysis, the new species *Scopuloidesfarinacea* was sister to *S. allantoidea* (Fig. 4), consistent with previous research (Gu et al. 2024). Morphologically, *Scopuloidesfarinacea* differs from *S. allantoidea* by having greyish, white to cream basidiomata, while the latter has larger basidia (9–12 × 3–4 μm; Chen et al. 2021). *Scopuloidesfarinacea* differs from *S. rimosa* by the latter having a greyish hymenial surface, larger basidia (10–12 × 3–4 μm), and larger lamprocystidia (40–50 × 8–10 μm; Jülich 1982).

A morphometric comparison of *Scopuloides* species reveals diagnostic features that characterize the newly recognized species *S. farinacea* (Table 4). Phylogenetic analysis also indicated that *S.yunnanensis* was nested in the genus *Scopuloides*.

**Table 4.** A morphological comparison between *Scopuloidesfarinacea* and ten similar species in the genus *Scopuloides*.

Species name	Hymenial surface	Generative Hyphae	Cystidia	Basidia	Basidiospores	References
*Scopuloidesallantoidea*	Ceraceous to pruinose, odontioid, not cracked, grayish, white to cream	Thick-walled, branched	Lamprocystidia, conical or subulate, 30–70 × 7–17 µm; septate cystidia, cylindrical, apically capitate, rounded or narrow, 5–13 µm diam, thick-walled	Cylindrical to clavate, 9–12 × 3–4 µm	Allantoid, 3.3–3.7 × 1.2–1.4 µm	Chen et al. (2021)
*Scopuloidesdimorpha*	Membranaceous, odontioid, not completely cracked, white, catridge buff, ivory buff or pale pinkish buff	Thin-walled, unbranched	Lamprocystidia, 35 × 5–17 µm; septate cystidia, cylindrical, fimbriatus apice, thin-walled	Subclavate to cylindrical, 15 × 3.5–3.8 µm	Ellipsoid, 3–3.2 × 1.2–1.8 µm	Lin and Chen (1990)
*Scopuloidesellipsoidea*	Ceraceous, grandinioid, yellowish white, gray, yellowish gray to orange gray	Thick-walled, rarely branched	Lamprocystidia, subconical to subfusiform, 18–30 × 4.5–11 µm	Subclavate to subcylindrical, 10–18 × 3–4 µm	Ellipsoid, 2.6–3.1 × 1.5–1.8 µm	Li et al. (2025)
***Scopuloidesfarinacea***	**Coriaceous, grandinoid, pale cream to buff**	**Thin to slightly thick-walled, branched**	**Lamprocystidia, conical or subulate, 21.2–39.5 × 6–13 µm**	**Subcylindrical, slightly flexuous, 4.6–8 × 1.8–2.7 µm**	**Ellipsoid, 2.8–3.5 × 1.4–2 µm**	**Present study**
*Scopuloidesgrandinioides*	Ceraceous, grandinioid, grayish orange	Thick-walled, rarely branched	Lamprocystidia, subulate to subfusiform, 45–55 × 7–14 µm; aculeal cystidia single, cylindrical, with several secondary septa, 52–90 × 6–12 µm, thick-walled	Subcylindrical, 10–15 × 3–4 µm	Allantoid to subcylindrical, 3–3.5 × 1.1–1.2 µm	Li et al. (2025)
*Scopuloideshydnoides*	Ceraceous, odontoid, distinctly cracked, whitish or grayish	Thick-walled, branched	Lamprocystidia, 40–60 × 8–12 µm; septate cystidia, cylindrical, Thick-walled	Subclavate, 12–15 × 3.5–4.5 µm	Short-allantoid, 3.5–4 × 1.8–2 µm	Hjortstam and Ryvarden (1979)
*Scopuloidesleprosa*	Membranaceous to subceraceous, smooth to irregular, rarely slightly grandinioid, deeply cracked; whitish to yellowish alutaceous, ochraceous	Thin to thick-walled, branched	Lamprocystidia, fusiform, 40–80 × 8–14 µm; cylindrical leptocystidia with obtuse or slightly swollen apex, 150 × 8–10 µm	Narrowly clavate, 20–30 × 4–6 µm	Ellipsoid, 4–5.5 × 2.5–3 µm	Boidin et al. (1993)
*Scopuloidesmagnicystidiata*	Cracking moderately, pale smoke gray to pale olive gray, older areas cartridge buff	Thick-walled, infrequently branched	Cylindrical with simple septa, often constricted at septa,150 × 20 µm, thin-walled	Clavate, 11–14 × 4–5.5 µm	Oblong to short-cylindric, 4–5 × 2–2.5 µm	Gilbertson and Nakasone (2003)
*Scopuloidesrimosa*	Ceraceous, odontoid, more or less grayish	Thin to thick-walled	Lamprocystidia, conical, 40–50 × 8–10 µm	Subclavate, 10–12 × 3–4 µm	Suballantoid, 3.5–4.5 × 1.5–2 µm	Jülich (1982)
*Scopuloidessubgelatinosa*	Ceraceous, widely hydnaceous to odontoid, cracks locally abundant, grayish brown, light brown to brown	Thick-walled, moderately branched	Pseudocystidia narrowly clavate with an obtuse apex or fusiform with an acute apex; 50–70 × 7–8 µm; encrusted cystidia, clavate to broadly fusiform with acute or rounded apices, 25–40 × 5.5–8 μm	–	Ellipsoid, 2.7–3 × 1.3–1.8 µm	Nakasone (2003)
*Scopuloidesyunnanensis*	Membranaceous, grandinoid, white to slightly cream	Thin to thick-walled, rarely branched	Lamprocystidia conical or subulate, 15–34 × 5–12 μm; septate cystidia, cylindrical, apically capitate, rounded or narrow, 18.5–40.5 × 4.5–7.5 μm	Cylindrical, 7–14.5 × 3–5 μm	Allantoid, 2.8–3.7 × 1.4–2 µm	Gu et al. (2024)

Wood-inhabiting fungi are very rich in Yunnan Province of China, and many new taxa have been recently described from the province (Dai et al. 2021; Wang et al. 2021; Wu et al. 2022b; Wang et al. 2023, 2024; Dong et al. 2023; Mao et al. 2023; Yang et al. 2023, 2024; Yuan et al. 2023; Zhang et al. 2023, 2024, 2025; Zhou et al. 2024, 2025; Liu et al. 2025b; Yang et al. 2025a). However, there are still unknown taxa of wood-inhabiting fungi in this region, and more new species could be discovered through further investigations in different areas of Yunnan Province.

## Supplementary Material

XML Treatment for Crystallicutis albomarginata

XML Treatment for Efibula glossophora

XML Treatment for Efibula punctata

XML Treatment for Scopuloides farinacea

## ﻿Additional information

### Use of AI

No use of AI was reported.

### Author ORCIDs

Zirui Gu https://orcid.org/0000-0003-1476-9040

Chunqin Zhou https://orcid.org/0009-0004-8117-1537

Jianling Zhang https://orcid.org/0009-0005-3268-7665

Yu Liu https://orcid.org/0009-0009-0049-9853

Qiaohua Deng https://orcid.org/0009-0002-4683-2702

Rongcong Yang https://orcid.org/0009-0006-0646-5265

Shunqiang Yang https://orcid.org/0009-0001-1058-9707

Changlin Zhao https://orcid.org/0000-0002-8668-1075

### Data availability

All of the data that support the findings of this study are available in the main text.
